# An enhancer variant at 16q22.1 predisposes to hepatocellular carcinoma via regulating *PRMT7* expression

**DOI:** 10.1038/s41467-022-28861-0

**Published:** 2022-03-09

**Authors:** Ting Shen, Ting Ni, Jiaxuan Chen, Haitao Chen, Xiaopin Ma, Guangwen Cao, Tianzhi Wu, Haisheng Xie, Bin Zhou, Gang Wei, Hexige Saiyin, Suqin Shen, Peng Yu, Qianyi Xiao, Hui Liu, Yuzheng Gao, Xidai Long, Jianhua Yin, Yanfang Guo, Jiaxue Wu, Gong-Hong Wei, Jinlin Hou, De-Ke Jiang

**Affiliations:** 1 State Key Laboratory of Organ Failure Research, Guangdong Key Laboratory of Viral Hepatitis Research, Guangdong Institute of Liver Diseases, Department of Infectious Diseases and Hepatology Unit, Nanfang Hospital, Southern Medical University, 510515 Guangzhou, China; 2 School of Life Sciences, Central South University, 510006 Changsha, China; 3 State Key Laboratory of Genetic Engineering, Collaborative Innovation Center for Genetics and Development, Human Phenome Institute, School of Life Sciences, Fudan University, 200438 Shanghai, China; 4 School of Public Health (Shenzhen), Sun Yat-sen University, 528406 Shenzhen, China; 5 Department of Epidemiology, Naval Medical University, 200433 Shanghai, China; 6 Institute of Bioinformatics, School of Basic Medical Science, Southern Medical University, 510515 Guangzhou, China; 7 School of Public Health, Fudan University, 200032 Shanghai, China; 8 School of Basic Medical Sciences; The Sixth Affiliated Hospital of Guangzhou Medical University, Qingyuan People′s hospital, Guangzhou Medical University, 510182 Guangzhou, China; 9 Department of Forensic Medicine, Medical College of Soochow University, 215123 Suzhou, Jiangsu Province China; 10 Department of Pathology, Youjiang Medical College for Nationalities, 533000 Baise, Guangxi Province China; 11 Biocenter Oulu, Faculty of Biochemistry and Molecular Medicine, University of Oulu, 90014 Oulu, Finland; 12 School of Basic Medical Sciences, Fudan University, 200032 Shanghai, China

**Keywords:** Hepatocellular carcinoma, Genome-wide association studies, Epigenetics

## Abstract

Most cancer causal variants are found in gene regulatory elements, *e.g*., enhancers. However, enhancer variants predisposing to hepatocellular carcinoma (HCC) remain unreported. Here we conduct a genome-wide survey of HCC-susceptible enhancer variants through a three-stage association study in 11,958 individuals and identify rs73613962 (T > G) within the intronic region of *PRMT7* at 16q22.1 as a susceptibility locus of HCC (OR = 1.41, *P* = 6.02 × 10^−10^). An enhancer dual-luciferase assay indicates that the rs73613962-harboring region has allele-specific enhancer activity. CRISPR-Cas9/dCas9 experiments further support the enhancer activity of this region to regulate *PRMT7* expression. Mechanistically, transcription factor HNF4A binds to this enhancer region, with preference to the risk allele G, to promote *PRMT7* expression. *PRMT7* upregulation contributes to in vitro, in vivo, and clinical HCC-associated phenotypes, possibly by affecting the p53 signaling pathway. This concept of HCC pathogenesis may open a promising window for HCC prevention/treatment.

## Abstract (web summary)

The role of enhancer variants in hepatocellular carcinoma (HCC) predisposition remains unknown. Here, the authors perform a genome-wide survey of HCC-susceptible enhancer variants in 11,958 individuals, identify rs73613962 within the intronic region of *PRMT7* and find that *PRMT7* upregulation predisposes to HCC.

## Introduction

Hepatocellular carcinoma (HCC) is one of the highly common malignant diseases and ranks the fourth leading cause of cancer-related deaths worldwide^1^. The interplay between genetic and environmental factors drives HCC development and progression. Chronic infections of hepatitis B virus (HBV) or hepatitis C virus (HCV), aflatoxin B1 exposure, and alcohol consumption are the known etiologies for HCC^2^. As for the genetic predisposition to HCC, our group and others have identified several HCC risk-associated single-nucleotide polymorphisms (SNPs) by genome-wide association studies (GWASs) over the past several years^3–10^. To date, however, the causality and underlying mechanisms of the HCC-susceptible SNPs identified remain poorly understood.

Recently, numerous studies on other cancers have provided lines of evidence that the causal SNPs usually reside in gene regulatory elements, for example, enhancers, which can modulate gene expression through long-distance genomic interactions^11–15^. For example, Gao et al. discovered that a GWAS-identified prostate cancer-susceptible SNP rs11672691 resides in an enhancer element and alters the binding site of transcription factor HOXA2. Due to the preferential binding of HOXA2 to the risk allele G of rs11672691, the transcriptional levels of two biologically plausible candidate genes, the long noncoding RNA (lncRNA) *PCAT19* and protein-coding gene *CEACAM21*, are elevated, and consequently promote prostate cancer cell growth and tumor progression^14^. In a similar study, the authors found that the prostate cancer-susceptible SNP rs11672691 mainly regulates *PCAT19* expression to induce prostate cancer by mediating the promoter-enhancer switch^15^. In the two studies, all demonstrated that the casual SNP resides in a regulatory element and affects the expression of the targeted gene(s) by altering the binding affinity of a certain transcription factor. Consequently, the regulated target genes further influence cancer initiation and progression. However, it remains unknown whether there are HCC-susceptible SNP(s) present in the regulatory elements, such as enhancers, and what are the underlying mechanisms by which such susceptible SNP(s) drive HCC development.

In this work, we find a HCC-associated susceptibility locus rs73613962 (T > G), which is resided in an enhancer located in the intronic region of *PRMT7* at 16q22.1. In the following series of functional experiments, we demonstrate that rs73613962 regulates the expression of its host gene *PRMT7* by affecting the binding preference of the transcription factor HNF4A to the alleles of rs73613962. Further, we investigate that upregulation of *PRMT7* contributes to HCC-related phenotypes possibly via p53 signaling pathway.

## Results

### Identification of a candidate enhancer variant rs73613962 at 16q22.1

**Fig. 1:**
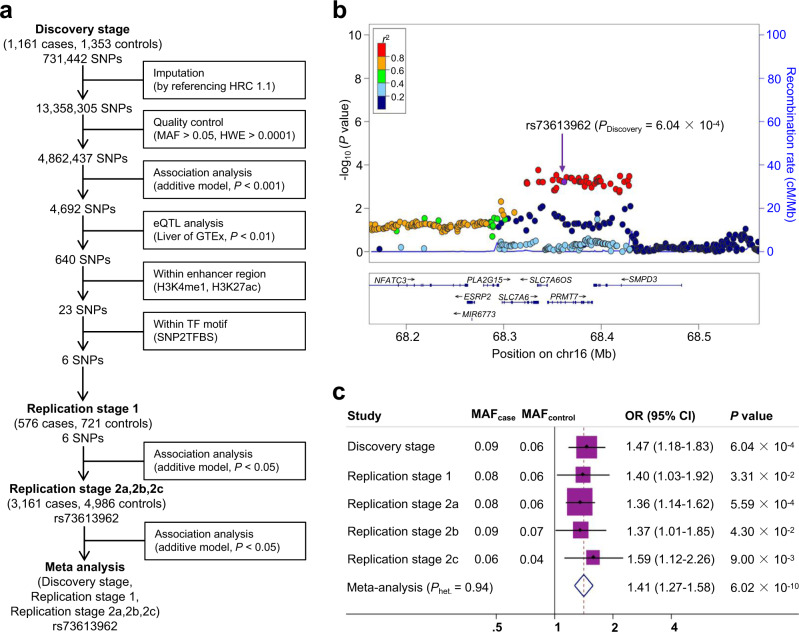
A candidate enhancer variant rs73613962 is screened and validated to be significantly associated with HCC risk. **a** Flowchart shows the candidate single-nucleotide polymorphisms (SNPs) in enhancers selected from the discovery stage and validated in two stages of replication. HRC Haplotype Reference Consortium, MAF minor allele frequency, HWE Hardy-Weinberg equilibrium, eQTL expression quantitative trait loci, TF transcription factor. **b** Regional association plot shows the association results (−log_10_ *P*) of all the SNPs in the region 100 kb upstream and 100 kb downstream of *PRMT7* rs73613962 in Discovery stage (*n* = 1161 cases, *n* = 1353 controls). The association of each SNP with HCC risk was evaluated through logistic regression under an additive model adjusting for gender and age. As the index SNP, rs73613962 is shown in purple, and the r^2^ values of the remaining SNPs are indicated by color. The genes within the region are annotated and shown as arrows. **c** The meta forest plot shows the association results of rs73613962 in the five independent populations of the discovery stage, replication stage 1, and replication stages 2a, 2b, and 2c. The association of rs73613962 with HCC risk in each population was calculated through logistic regression under an additive model adjusting for gender and age. The MAFs in cases and controls are shown for each population. The ORs and 95% CIs were calculated by considering the major allele as a reference. The center of each square and the horizontal line show the OR and the corresponding 95% CI, respectively. The pooled OR was obtained using the fixed-effects model and is represented by a hollow diamond, where its center indicates the OR and its ends correspond to the 95% CI.

To harvest HCC risk-associated SNPs residing in enhancer elements, we conducted a screening process of our pre-existing GWAS data^5^ and then a two-stage validation study, totaling 4898 HCC cases and 7060 non-HCC controls (Fig. 1a, Supplementary Table 1).

In the discovery stage, we screened candidate enhancer variants across the whole genome through a series of bioinformatical analyses. First, we did association analyses for over 4.8 million SNPs from the GWAS data after whole-genome imputation and quality control, and SNPs with *P* < 0.001 were retained for the next steps (Supplementary Data 3). Then, we screened SNPs that potentially change enhancer activity by the following criteria: (1) the SNPs are significantly associated with gene expression through *cis*-expression quantitative trait loci (eQTL) analysis using the Genotype-Tissue Expression (GTEx) liver tissue data^16^; (2) the SNPs are located in the enhancer-associated histone modification regions (monomethylation at histone H3 lysine 4 (H3K4me1), and acetylation of histone H3 at lysine 27 (H3K27ac)) in the liver hepatoma cell line HepG2^17^; (3) the SNPs are within the binding motif of transcription factors as transcription factor binding is usually required for enhancer activity^18^. After the screening process in the discovery stage, six candidate SNPs were obtained for validation in the following two stages of replication (Fig. 1a).

At replication stage 1, the six screened candidate SNPs were assessed for association with HCC risk, and only rs73613962 (T > G), located in the intronic region of *PRMT7* at 16q22.1 (Fig. 1b), was validated (*P* < 0.05, and with the same direction of association as in the discovery stage, the minor allele G is the risk allele, whereas the major allele T is the non-risk allele; Fig. 1c, Supplementary Table 2).

At replication stage 2, which included three independent subject sets, rs73613962 was consistently validated to be significantly associated with HCC risk (*P* < 0.05 in each subject set, and with the same direction of association as in the discovery stage and the replication stage 1; Fig. 1c).

In a joint analysis of the three stages, rs73613962 was identified as a HCC susceptibility locus at a GWAS significant level (OR = 1.41, 95% CI = 1.27–1.58, *P* = 6.02 × 10^−10^; Fig. 1c) after a Bonferroni multiple-test correction (a significance threshold of ~1.04 × 10^−8^). We next sought to explore its biological mechanism and significance by integrating a series of analyses and experiments.

### The rs73613962-containing region has allele-specific enhancer activity

**Fig. 2:**
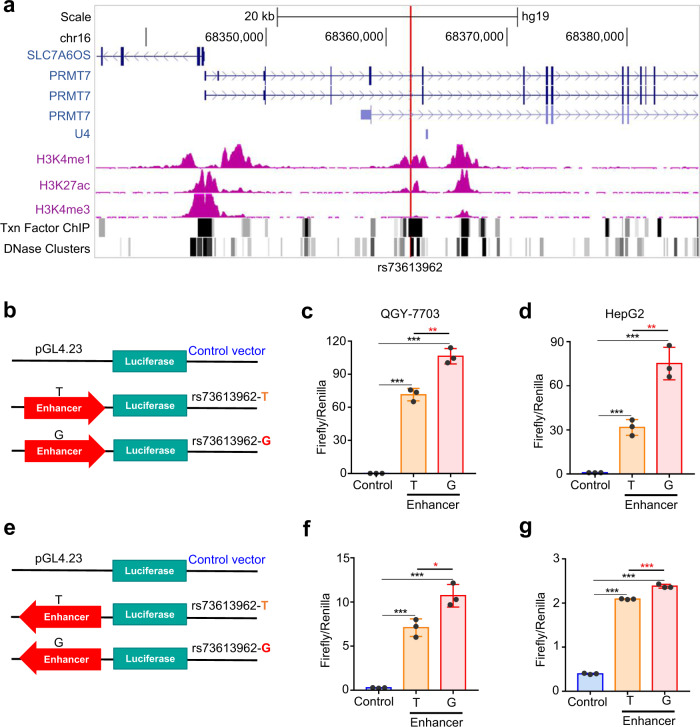
The rs73613962-containing region has an enhancer signal and allele-specific enhancer activity. **a** Overview of the H3K4me1, H3K27ac, and H3K4me3 chromatin modifications, the binding sites of transcription factors (Txn Factor ChIP), and DNase cluster distribution in the regions surrounding rs73613962 of the HepG2 cell line supported from the UCSC genome browser. rs73613962 is indicated by the red vertical line. **b**–**g** The dual-luciferase assays of enhancer activity for the empty vector (Control), and the plasmids with the insertion of the rs73613962-centered region (Enhancer-T and Enhancer-G). Luciferase activity is individually examined when the rs73613962-centered region is inserted in the luciferase vector in the forward (**b**–**d**) or reverse orientation (**e**–**g**). The assays are performed in two HCC cell lines, QGY-7703 (**c**, *P* < 0.0001 in Enhancer-T or Enhancer-G compared to Control, and *P* = 0.0026 in Enhancer-G compared to Enhancer-T; **f**, *P* = 0.0003 and *P* = 0.0001 in Enhancer-T or Enhancer-G compared to Control, and *P* = 0.0181 in Enhancer-G compared to Enhancer-T) and HepG2 (**d**, *P* = 0.0006 and *P* = 0.0003 in Enhancer-T or Enhancer-G compared to Control, and *P* = 0.0036 in Enhancer-G compared to Enhancer-T; **g,** *P* < 0.0001 in Enhancer-T or Enhancer-G compared to Control, and *P* = 0.0003 in Enhancer-G compared to Enhancer-T). Values are expressed as the mean ± SD, *n* = 3 in **c**, **d**, **f**, and **g**. ** and *** mean *P*-values less than 0.01 and 0.001, respectively (two-sided student’ *t*-test*)*.

To confirm that the rs73613962-containing region has the features of an enhancer, we examined three more chromatin signals derived from the Encylopedia of DNA Elements (ENCODE, the ENCODE Project Consortium)^19, 20^ datasets in UCSC genome browser^21^ (trimethylation at histone H3 lysine 4 (H3K4me3), transcription factor chromatin immunoprecipitation (ChIP) followed by deep sequencing (ChIP-seq), and DNase I hypersensitive site), in addition to H3K4me1 and H3K27ac. The obvious epigenetic signals of H3K4me1 and H3K27ac near the rs73613962 locus strongly support the presence of enhancer activity in the rs73613962-containing region in HepG2 cells (Fig. 2a). The high signals of transcription factor binding and DNase clusters near rs73613962 reinforce the probability of the existence of enhancer activity (Fig. 2a). We next examined the enhancer activity of the rs73613962-containing region in other cell lines. Except for HepG2, most of the other cell lines in this region do not have the signals of enhancer-specific chromatin modification (H3K4me1 and H3K27ac), or the signals are very low, implying that the rs73613962-containing region is a strong enhancer in the liver cell line (Supplementary Fig. 1).

To experimentally validate the enhancer activity of the rs73613962-containing region, we performed an enhancer dual-luciferase reporter assay and found that the rs73613962-centered DNA sequence showed higher luciferase activity than the control sequence in both the forward and reverse directions in HCC cell lines (Fig. 2b–g, Supplementary Fig. 2). We next examined whether rs73613962 could affect enhancer activity. The results demonstrated that the sequence with the risk allele G showed significantly higher luciferase activity than that with the non-risk allele T in these HCC cell lines (Fig. 2b–g, Supplementary Fig. 2), suggesting that this region may function as an enhancer with allele-specific activity.

In sum, these data indicate that the *PRMT7* intronic region containing the HCC-susceptible variant rs73613962 has allele-specific enhancer activity, and the risk allele G of rs73613962 corresponds to higher enhancer activity as compared to the non-risk allele T.

### The enhancer variant rs73613962 modulates *PRMT7* expression

**Fig. 3:**
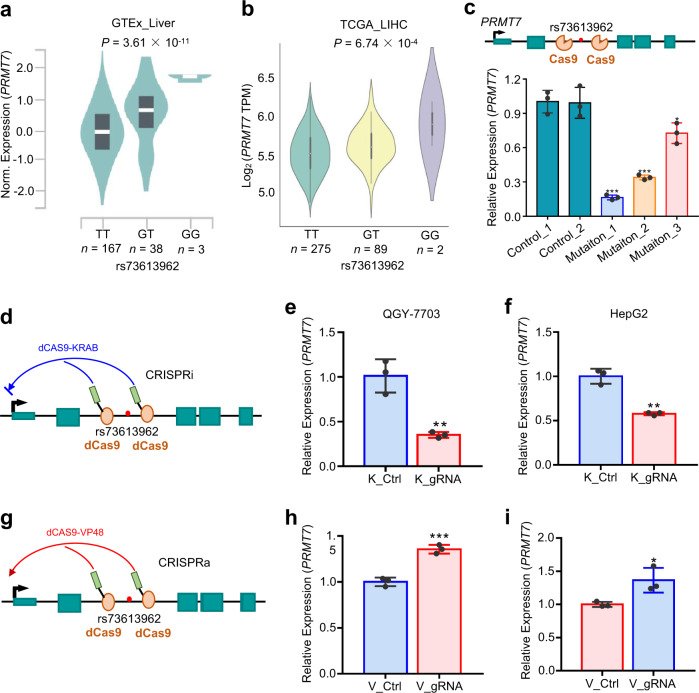
The enhancer variant rs73613962 modulates *PRMT7* expression by regulating enhancer activity. **a**, **b** The association between the genotypes of rs73613962 and *PRMT7* expression through linear regression analysis in GTEx liver tissues (**a**, the center white line and the black box in the plot indicate the median and the first to third quartile, respectively) and TCGA LIHC (**b**, the center white dot, the black limit, and the whisker represent the median, the first to third quartile, and the 95% confidence interval, respectively). **c** Gene editing of the region surrounding rs73613962 by CRISPR-Cas9. The above panel shows the basic principle of the Cas9 assay. The Cas9 protein binds to the neighboring sequence of the rs73613962 site guided by small guide RNAs (sgRNAs) targeting the upstream and downstream of this locus to induce the double-stranded breaks of these regions in the *PRMT7* gene. qPCR detection of relative *PRMT7* expression upon the controls without any mutation in these regions (Control_1, 2), and the mutations being edited in these regions (Mutation_1, 2, 3) are shown in the bottom panel (*P* = 0.0001, *P* = 0.0003, and *P* = 0.0231 in Mutation_1, 2, or 3 compared to Control_1, respectively). **d**, **g** Illustration of the dCas9 assay. The above diagram shows the CRISPR-interference (CRISPRi) assay. DCas9-KRAB binds to the upstream and downstream of rs73613962 guided by sgRNAs to inhibit *PRMT7* expression (**d**). The bottom diagram shows the CRISPR-activation (CRISPRa) assay to activate the expression of *PRMT7* guided by sgRNAs (**g**). **e**, **f** RNA level of *PRMT7* detected by qPCR in the CRISPRi assay in the QGY-7703 (**e**, *P* = 0.0038 in dCas9-KRAB_gRNA (K_gRNA) compared to dCas9-KRAB_Control (K_Ctrl)) and HepG2 (**f**, *P* = 0.0011 in K_gRNA compared to K_Ctrl) cell lines. **h**, **i** RNA level of *PRMT7* detected by qPCR in the CRISPRa assay in the QGY-7703 (**h**, *P* = 0.0007 in dCAS9-VP48_gRNA (V_gRNA) compared to dCAS9-VP48_Control (V_Ctrl)) and HepG2 (**i**, *P* = 0.0300 in V_gRNA compared to V_Ctrl) cell lines. Values are expressed as the mean ± SD, *n* = 3 in **c**, **e**, **f**, **h**, and **i**. *, **, and *** mean *P*-values less than 0.05, 0.01, and 0.001, respectively (two-sided student’ *t*-test).

To explore whether rs73613962 is associated with the expression of its nearby genes, *cis*-eQTL analysis was performed in liver tissues from the GTEx Project, and rs73613962 was discovered to be significantly associated only with the expression level of *PRMT7* in one mega-base pair window that includes 36 genes (Fig. 3a, Supplementary Data 1). Interestingly, *PRMT7* is the host gene of rs73613962 (Fig. 2a), implying that this intronic enhancer may directly regulate the transcription of *PRMT7*. Remarkably, the risk allele G of rs73613962 was found to be strongly associated with increased expression of *PRMT7* (Fig. 3a), consistent with the above luciferase assay results (Fig. 2b–g). This finding was also replicated in all the liver hepatocellular carcinoma (LIHC) subjects, tumor, and normal specimens of The Cancer Genome Atlas (TCGA) Program by analyzing the genotype of rs73613962 and the expression data of *PRMT7* (Fig. 3b, Supplementary Fig. 3a, b). Furthermore, the significant association of rs73613962 with the expression level of *PRMT7* was consistent in subgroups of TCGA-LIHC subjects stratified by the status of HBV infection or ethnicity (Asians vs. Europeans; Supplementary Fig. 3c–f), indicating that it may be independent of HBV infection and ethnicity.

To examine whether the rs73613962-containing region plays a causal role in *PRMT7* expression, in *cis* mutation, repression, and activation by CRISPR-Cas9, dCAS9-KRAB (CRISPR-interference, CRISPRi), and dCAS9-VP48 (CRISPR-activation, CRISPRa) assays, respectively, were performed in HCC cell lines, with small guide RNAs (sgRNAs) targeting the region surrounding rs73613962 (Fig. 3c, d, g). In the CRISPR-Cas9 experiment, three single clones with different mutations at the rs73613962 locus and two clones without any mutation in this region were selected for the following investigation. As shown in Fig. 3c, a significantly decreased expression of *PRMT7* was detected in the three mutated clones as compared to the two controls, suggesting a direct regulation of the rs73613962-harboring region on *PRMT7* expression. Consistent with this result, the CRISPRi assay showed that the enhancer activity of the rs73613962-containing region may be attenuated when sgRNAs targeted the upstream and downstream sequences of rs73613962, as reflected by the decreased expression of *PRMT7* in both the QGY-7703 and HepG2 cell lines (Fig. 3d–f, Supplementary Fig. 4a, b). In the CRISPRa assay, we detected that the mRNA and protein levels of *PRMT7* were significantly elevated in both the QGY-7703 and HepG2 cell lines (Fig. 3g–i, Supplementary Fig. 4c, d), indicating that the enhancer activity of this region may be increased by the dCAS9-fused transcription activation domain VP48.

Since the stable RNA level is determined by the rates of RNA transcription and degradation, we next performed a nascent-RNA capturing experiment to further demonstrate that *PRMT7* expression was regulated at the transcriptional level by the rs73613962-harboring region. As indicated in Supplementary Fig. 5, the alteration of *PRMT7* abundance occurred at the nascent-RNA level, with a significant decrease in the CRISPRi assay and an obvious increase in the CRISPRa assay.

Collectively, these results suggest that the intronic enhancer variant rs73613962 modulates the expression of its host gene *PRMT7* by influencing enhancer activity.

### The risk allele G of rs73613962 elevates the binding ability of HNF4A

**Fig. 4:**
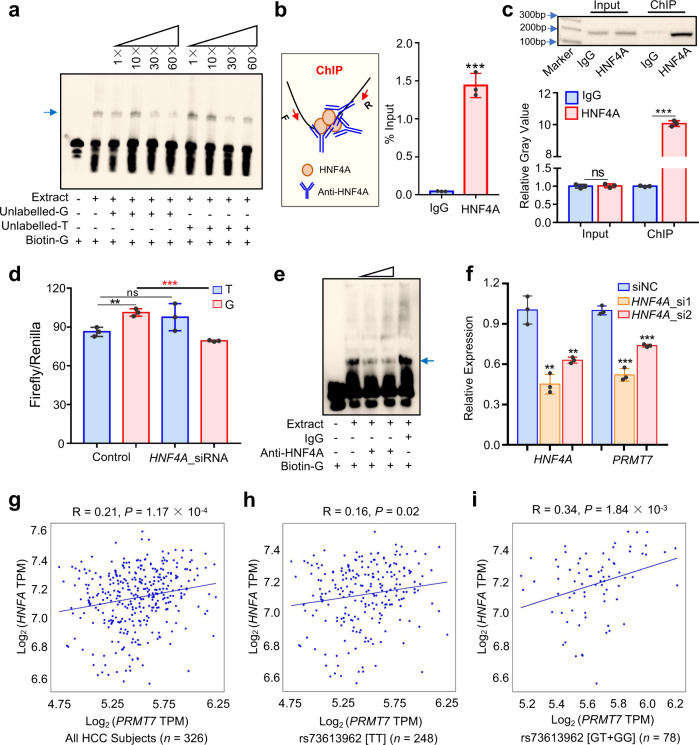
The risk allele G of rs73613962 enhances the binding ability of transcription factor HNF4A to promote *PRMT7* expression. **a** The result of the competitive EMSA. The binding affinity of protein to DNA oligos is demonstrated by adding unlabeled-G or unlabeled-T to the reaction. The light blue arrow indicates the protein-biotin-G-oligo complexes. This experiment is replicated two times at least and similar results are observed. **b** The enrichment of HNF4A on the rs73613962-containing region examined by ChIP. The left panel shows the diagram for HNF4A-ChIP. The antibody of HNF4A recognizes and binds to HNF4A; thus, the DNA sequence bound by HNF4A is pulled down from the fragmented chromatin. Then, the enriched DNA sequence is detected by the following qPCR as shown in the right panel. *P* = 0.0001 in HNF4A compared to IgG. **c** ChIP-PCR results. The agarose gel electrophoresis of PCR products for Input and ChIP, respectively, in the above panel. Light blue arrows indicate the marker bands. Source data are provided as a Source Data file. The relative gray value of each PCR band is analyzed using ImageJ in the bottom panel. *P* = 0.7769 and *P* < 0.0001 in HNF4A compared to IgG in Input and ChIP, respectively. **d** The dual-luciferase assay of enhancer activity for the enhancer-T and enhancer-G before and after the downregulation of *HNF4A*. *P* = 0.0046 in Enhancer-G compared to Enhancer-T without *HNF4A* knockdown, and *P* = 0.1493 and *P* = 0.0002 in Enhancer-T or Enhancer-G with *HNF4A* knockdown to Enhancer-T or Enhancer-G without *HNF4A* knockdown, respectively. **e** The result of the blocking EMSA of HNF4A. The amount of anti-HNF4A antibody in lane 3 and lane 4 is 1 μg and 2 μg, respectively. The IgG amount in lane 5 is 2 μg. The light blue arrow indicates the HNF4A-biotin-G-oligo complexes. We repeat this experiment two times at least and always observe the similar results. **f** qPCR detection of *HNF4A* and *PRMT7* expressions after the downregulation of *HNF4A* in cells through two siRNAs. For detection of *HNF4A* expression level, *P* = 0.0017 and 0.0037 in HNF4A_small interfering RNA (HNF4A_si)1 or HNF4A_si2 compared to small interfering RNA negative control (siNC), respectively. For detection of *PRMT7* expression level, *P* = 0.0001 and *P* = 0.0002 in HNF4A_si1 or HNF4A_si2 compared to siNC, respectively. All the above experiments were conducted in the QGY-7703 cell line (**a**–**f**). **g**–**i** The correlation of *PRMT7* and *HNF4A* expressions showed in all HCC subjects (**g**), and subjects with the rs73613962 TT homozygote (**h**), or rs73613962 G allele carriers (**i**) in TCGA LIHC. TPM, transcripts per million. Values are expressed as the mean ± SD, *n* = 3 in **b**–**d**, and **f**. ‘ns’ means not significant; ** and *** mean *P* values less than 0.01, and 0.001, respectively (two-sided student’ *t*-test).

As the risk allele G of rs73613962 is associated with higher expression of the enhancer target gene *PRMT7*, we speculated that the allele G is more likely to recruit enhancer-associated transcription factor than the allele T. To test this, we first performed an electrophoretic mobility shift assay (EMSA) in the QGY-7703 cell line and found that the sequence with the allele G had a higher binding affinity to the nuclear extract than the sequence with the allele T (Supplementary Fig. 6a). In the competitive binding experiment, we further observed that the oligo with the allele G had a higher competitive binding ability to the nuclear extract than that of the oligo with the allele T (Fig. 4a, Supplementary Fig. 6b). Such a conclusion remained true in the HepG2 cell line (Supplementary Fig. 7). These findings indicated that the rs73613962-containing sequence may act as a regulatory element by recruiting certain transcription factors, possibly with binding preference to the risk allele G over the non-risk allele T. To determine which transcription factor in the nuclear extract is correlated with allele-specific enhancer activity, we first screened transcription factors that potentially bind to the region surrounding rs73623962 using publicly available ChIP-seq datasets and related databases, and we found that HNF4A, which is one of the important hepatocyte nuclear factors, may be the main transcription factor affected by rs73613962.

We first confirmed whether HNF4A binds to the rs73613962-resided enhancer by carrying out a ChIP experiment on the rs73613962-containing sequence. As shown in Fig. 4b, a strong enrichment of HNF4A binding on this region was detected by ChIP coupled with quantitative polymerase chain reaction (ChIP-qPCR) in the QGY-7703 cell line, when the HNF4A-specific antibody was compared to the control antibody immunoglobulin G (IgG). Such a result was replicated in the HepG2 cell line (Supplementary Fig. 8a). While no significant enrichment of HNF4A on the downstream region of rs73613962 was observed when compared HNF4A-specific antibody to the control antibody IgG (Supplementary Fig. 9). Additionally, ChIP coupled with polymerase chain reaction (ChIP-PCR) also confirmed the binding between HNF4A and the rs73613962-containing enhancer region in both the QGY-7703 and HepG2 cell lines (Fig. 4c, Supplementary Fig. 8b, c). These lines of evidence indicated a direct binding between HNF4A and this intronic enhancer. Next, we transfected the vector having the insertion of G or T-centered sequence into the cells, and then conducted the HNF4A-ChIP. The ChIP-qPCR result showed that there was higher HNF4A enrichment in cells transfected with a vector having G-containing sequence compared to the empty vector and even the vector with T-containing sequence (Supplementary Fig. 10), partially demonstrated that HNF4A have a preferential binding to the risk allele G over the non-risk allele T.

As HNF4A binds more to the allele G, the knockdown of HNF4A is expected to have a greater impact on the allele G-containing enhancer activity. Using the enhancer dual-luciferase reporter assay, we observed that the luciferase activity of the allele G-containing enhancer was significantly decreased when compared to the control upon the downregulation of *HNF4A* by siRNA. In contrast, the luciferase activity of the allele T-containing enhancer was nearly unchanged upon *HNF4A* knockdown (Fig. 4d). Furthermore, adding anti-HNF4A to the nuclear extract ablated the binding between HNF4A and the allele G-containing sequence in both HCC cell lines (Fig. 4e, Supplementary Fig. 11).

As rs73613962 is associated with *PRMT7* expression, we speculated that the downregulation of a transcription factor will reduce the expression of the target gene *PRMT7* if this transcription factor is required for rs73613962-related enhancer-binding. We knocked down HNF4A in both the QGY-7703 and HepG2 cell lines and found decreased expression of *PRMT7* in these cells (Fig. 4f, Supplementary Fig. 12). Additionally, we found that the *PRMT7* RNA level was positively correlated with the expression of *HNF4A*, as shown by TCGA LIHC and liver tissues of GTEx (Fig. 4g, Supplementary Fig. 13). Intriguingly, a higher correlation was observed in subjects carrying with the G allele than those with the TT homozygote (Fig. 4h, i).

Together, these findings suggest that transcription factor HNF4A may play a key role in controlling the enhancer activity of the rs73613962-centered region, and the risk allele G of rs73613962 promotes a higher expression of *PRMT7* through preference binding of HNF4A to the risk allele G over the non-risk allele T.

### PRMT7 promotes cancer-related phenotypes

**Fig. 5:**
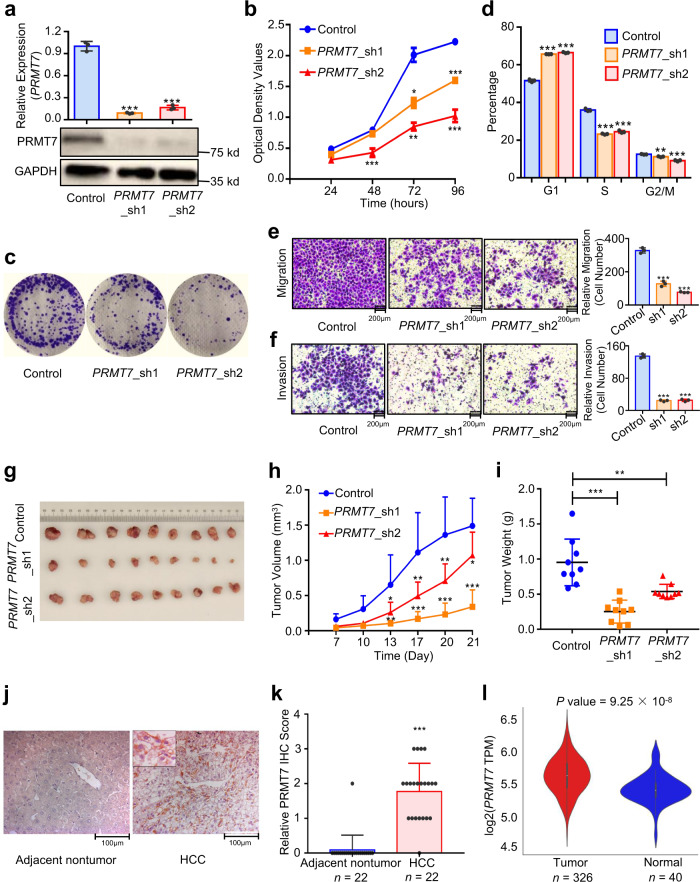
*PRMT7* downregulation reduces cancer-related phenotypes, and *PRMT7* is upregulated in HCC tumors. **a** qPCR and western blot detection of RNA and protein abundance of PRMT7 after *PRMT7* knockdown in cells by two shRNAs, respectively. *P* < 0.0001 in *PRMT7*_sh1 or *PRMT7*_sh2 compared to Control. Source data are provided as a Source Data file. **b** CCK-8 assay for *PRMT7*-depleted cells and the control. *P* < 0.0001 in *PRMT7*_sh1 or *PRMT7*_sh2 compared to Control at 96 h post-seeding. **c** Cell colony formation assay for cells with *PRMT7* knockdown and the control. **d** Cell cycle analysis of the control and *PRMT7*-downregulated cells. *P* < 0.0001 in *PRMT7*_sh1 or *PRMT7*_sh2 compared to Control in G1 and S phase. *P* = 0.0019 and *P* = 0.0002 in *PRMT7*_sh1 or *PRMT7*_sh2 compared to Control in G2/M phase, respectively. **e, f** The detection of cell migration (**e**, *P* = 0.0001 and *P* < 0.0001 in *PRMT7*_sh1 or *PRMT7*_sh2 compared to Control) and invasion (**f**, *P* < 0.0001 in *PRMT7*_sh1 or *PRMT7*_sh2 compared to Control) abilities of cells with *PRMT7* knockdown and the control. All the above experiments were conducted in the QGY-7703 cell line (**a**–**f**). **g**–**i** Xenograft experiment for *PRMT7* downregulation. The image (**g**), growth rate (**h**, *P* <0.0001 and *P* = 0.0263 in *PRMT7*_sh1 or *PRMT7*_sh2 compared to Control at 21 days after the injection of cells), and weight (**i**, *P* < 0.0001 and *P* = 0.0026 in *PRMT7*_sh1 or *PRMT7*_sh2 compared to Control) of the xenograft tumor are shown. **j** The IHC staining results of PRMT7 in adjacent non-tumor and HCC tissues. This staining is performed in 22 HCC patients with paired HCC tissues and non-tumor tissues. **k** The relative PRMT7 IHC score compared HCC tissues with adjacent non-tumor tissues. *P* < 0.0001 in HCC compared to adjacent non-tumor. **l** The expression of *PRMT7* in tumor specimens and normal tissues of TCGA LIHC. Values are expressed as the mean ± SD, *n* = 3 in **a**, **b**, and **d**–**f**. Values are expressed as the mean + SD, *n* = 9 in **h**, *n* = 22 in **k**. Values are expressed as the median with interquartile range in **i**, *n* = 9. *, **, and *** mean *P-*values less than 0.05, 0.01, and 0.001, respectively (two-sided student’ *t*-test).

We have demonstrated that the HCC risk allele G of rs73613962 inside the intron of target gene *PRMT7* enhanced the binding of HNF4A, and HNF4A in turn promoted the expression of *PRMT7*. We therefore asked whether the elevated expression of *PRMT7* would contribute to cancer-associated cellular phenotypes and thus lead to high HCC risk. Knocking down *PRMT7* by two short hairpin RNAs (shRNAs) both reduced the cell proliferation rate in QGY-7703 cells, as evaluated by the CCK-8 assay (Fig. 5a, b). *PRMT7*-downregulated cells also showed less colony formation ability than the control cells (Fig. 5c). Additionally, cells with depleted *PRMT7* expression showed an obvious G1-phase arrest and a significant decrease in the S phase as compared to the control cells (Fig. 5d). Furthermore, *PRMT7* knockdown led to significantly reduced cell migration and invasion ability in the HCC cell line QGY-7703 (Fig. 5e, f). Similar phenotypic changes were detected in the CRISPRi-edited QGY-7703 cell line (Supplementary Fig. 14). Additionally, the other four HCC cell lines with downregulation of *PRMT7* also showed reduced cell malignant phenotypes compared to the control, supporting the results detected in QGY-7703 (Supplementary Figs. 15–18).

An in vivo tumorigenicity assay further showed that *PRMT7* downregulation could decrease xenograft growth (Fig. 5g–i). Besides, overexpressing *PRMT7* increased the cell growth rate, cell migration, and invasion ability (Supplementary Fig. 19), reinforcing the functional speculation of *PRMT7* by the knockdown strategy. Supporting these above results, *PRMT7* was found to be more highly expressed in HCC tissues than that in adjacent non-tumor tissues examined by immunohistochemistry (IHC) (Fig. 5j, k). Furthermore, tumor specimens showed relatively higher *PRMT7* expression than normal tissues in TCGA LIHC (Fig. 5l). In addition, patients with high *PRMT7* expression showed suggestively significantly worse overall survival (*P* = 0.1) and significantly worse relapse-free survival than those with low *PRMT7* expression in HCC patients of TCGA LIHC (*P* = 0.002, Supplementary Fig. 20).

Taken together, we provided evidence to support that *PRMT7*, whose expression is regulated by the rs73613962-harboring intronic enhancer, can explain, at least in part, the association between rs73613962 and HCC risk.

### PRMT7 regulates the p53 signaling pathway through H4R3me2s modification

**Fig. 6:**
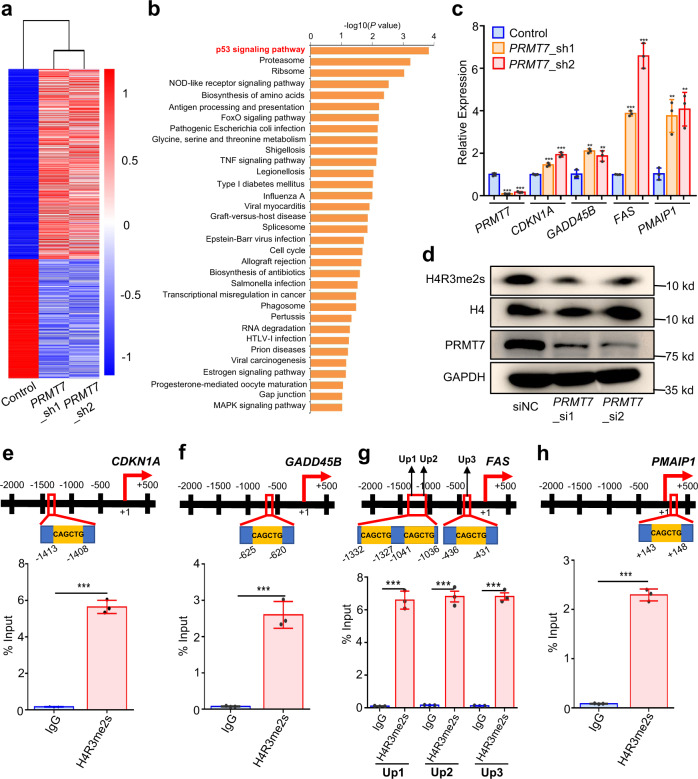
The p53 signaling pathway is regulated by PRMT7 in HCC cells. **a** HeatMap of the differentially expressed genes was analyzed using RNA-seq data after *PRMT7* knockdown in QGY-7703 cells. **b** Functional enrichment of the differentially expressed genes for KEGG was analyzed using DAVID bioinformatics resources. The p53 signaling pathway is highlighted in red. **c** qPCR detection of RNA level of four genes enriched in the p53 signaling pathway before and after *PRMT7* knockdown in QGY-7703 cells, respectively. *PRMT7*, *P* < 0.0001; *CDKN1A, P* = 0.0009 and *P* = 0.0001; *GADD45B*, *P* = 0.0011 and *P* = 0.0093; *FAS*, *P* < 0.0001; *PMAIP*, *P* = 0.0046 and *P* = 0.0033, in *PRMT7*_sh1 or *PRMT7*_sh2 compared to Control. **d** Western blot detection of H4R3me2s signal in the control and *PRMT7*-downregulated samples in QGY-7703 cell line. Source data are provided as a Source Data file. This experiment is replicated two times at least and similar results are observed. **e**–**h** The enrichment of H4R3me2s modification on the promoter region of four genes, *CDKN1A* (**e**), *GADD45B* (**f**), *FAS* (**g**), and *PMAIP1* (**h**), in the p53 signaling pathway was detected by ChIP-qPCR. Up1, Up2, and Up3 are the three sequences with CAGCTG motif in the promoter region of *FAS*. *P* < 0.0001 in HNF4A compared to IgG in **e**–**h**. Values are expressed as the mean ± SD, *n* = 3 in **c** and **e**–**h**. *, **, and *** mean *P*-values less than 0.05, 0.01, and 0.001, respectively (two-sided student’ *t*-test).

To explore the mechanism by which PRMT7 regulates HCC cell malignant phenotypes, we performed RNA-sequencing (RNA-seq) on QGY-7703 cells before and after *PRMT7* knockdown to identify the affected signaling pathways. Through function enrichment analysis of the differentially expressed genes by the Database for Annotation, Visualization and Integrated Discovery (DAVID), we found that the p53 signaling pathway which plays an important role in cancer development ranked the top 1, implying that these genes enriched in this pathway may be involved in PRMT7-mediated HCC development (Fig. 6a, b). We verified some genes, i.e., *CDKN1A*, *GADD45B*, *FAS,* and *PMAIP1*, belonging to the p53 signaling pathway by quantitative real-time polymerase chain reaction (qRT-PCR) (Fig. 6c).

It has been reported that the monomethylarginine (MMA) at arginine of histone 4 primed by PRMT7 is catalyzed to establish a repressive histone mark, a symmetrical dimethylation (SDMA) at arginine 3 in histone 4 (H4R3me2s)^22^. Next, we examined the signal of H4R3me2s modification upon *PRMT7*-downregulated cells and the control through Western blot. Significantly reduced H4R3me2s signal was detected but no obvious change was observed for H4 as compared the *PRMT7*-depleted samples to the control (Fig. 6d and Supplementary Fig. 21), indicating the regulation of PRMT7 on H4R3me2s modification of genes.

According to the published report, CAGCTG motif was the significantly enriched sequence in H4R3me2s binding regions^23, 24^. Therefore, we scanned the core promoter of these genes enriched in the p53 signaling pathway for the distribution of CAGCTG motif. Intriguingly, most of these genes′ promoter contained one or more CAGCTG motif. Next, to deeply explore the mechanism of the p53 signaling pathway involved in PRMT7-inducing cancer-related phenotypes, we performed H4R3me2s ChIP in the QGY-7703 cell lines, and then detected the enrichment of H4R3me2s on the CAGCTG motif-resided regions in the promoter of genes by qPCR. Here, we investigated the four genes which were upregulated in *PRMT7*-downregulated cells implicated by RNA-seq data and verified by qRT-PCR. As showed by Fig. 6e–h, the CAGCTG-containing region in the promoter of the four genes had significant enrichment of H4R3me2s compared to IgG.

Thus, these results together imply that the genes enriched in the p53 signaling pathway whose modification of H4R3me2s were regulated by PRMT7 may be involved in PRMT7-mediated cancer-related phenotypes.

## Discussion

**Fig. 7:**
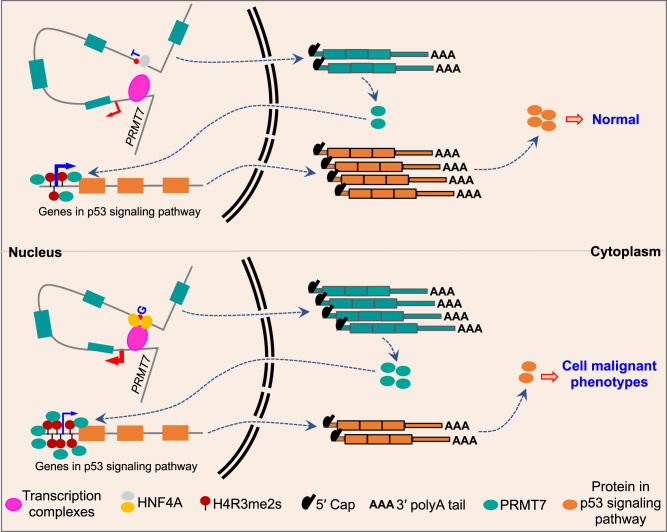
Model for the functional mechanism of the HCC risk-associated enhancer variant rs73613962 in the intronic region of *PRMT7*. The region containing the HCC susceptibility locus rs73613962 inside the intronic region of *PRMT7* is identified as an enhancer that can regulate *PRMT7* expression through the interaction with its promoter. Transcription factor HNF4A prefers to bind to the risk allele G over the non-risk allele T, which leads to the upregulated expression of *PRMT7*. Under this condition, the H4R3me2s modification on the promoter region of the genes in the p53 signaling pathway is elevated; thereby, the RNA and protein level is reduced. As a result, cells are induced to have enhanced malignant phenotypes, with higher proliferation rate and migration and invasion abilities.

HCC is the sixth most common and fourth most deadly cancer in the world^1^. Multiple susceptible genetic variants associated with HCC pathogenesis have been discovered using GWAS by both our group and other researchers over the past few years^3–10^. However, it still remains largely undefined whether these HCC risk-associated loci are the real casual SNPs, and if so, how do they play their roles in HCC? In this study, we identify a HCC risk-associated SNP rs73613962, which is located in the intronic enhancer region of *PRMT7* at 16q22.1. We find that the risk allele G of rs73613962 corresponds to higher enhancer activity, which promotes the expression of its host gene *PRMT7*. Mechanistically, transcription factor HNF4A prefers to bind to the risk allele G-containing enhancer sequence and promotes *PRMT7* expression, which ultimately leads to cancer-related phenotypes via the modification of H4R3me2s to regulate p53 signaling pathway (Fig. 7). This study reveals that a genetic variant inside an intronic enhancer could lead to HCC-associated phenotypes by affecting its host gene′s expression, largely extending the mechanistic understanding of HCC.

As one of the gene regulatory elements, enhancers can positively regulate gene expression, independent of genomic distance and orientation through a long-range interaction with target genes^18^. Due to the presence of distinct histone marks and transcription co-activators, enhancers scattered across the whole genome are easily distinguished from other regulatory DNA elements. According to previous reports, the complex disease risk-associated SNPs identified using GWAS commonly reside in the noncoding regions that usually overlap with putative regulatory elements, such as enhancer^25–28^. In this study, by focusing on the SNPs residing in the noncoding regions with enhancer activity, we indeed obtain a few candidate SNPs within the enhancer region through a genome-wide screening of our pre-existing GWAS data^5^. In the following independent validation populations, rs73613962 is confirmed as a significant susceptibility locus of HCC (OR = 1.41, 95% CI = 1.27–1.58, *P* = 6.02 × 10^−10^), which leads us to deeply examine the biological significance and molecular mechanism of this SNP in terms of enhancer regulation.

Multiple lines of evidence support the fact that rs73613962 is located in an enhancer region. First, it has the considerable signals of H3K4me1 and H3K27ac around, typical histone modifications of enhancer (Fig. 2a). Second, multiple transcription factors could potentially bind there, as supported by ENCODE ChIP-seq and DNase Clusters (Fig. 2a). Third, the enhancer luciferase assay shows a high activity of cloned sequences and the risk allele G had even higher activity than the non-risk allele T (Fig. 2b–d), consistent with the *cis*-eQTL results (Fig. 3a, b). Fourth, the manipulation of the sequence around rs73613962 does affect the expression of the host gene *PRMT7* (Fig. 3c–i). Fifth, liver transcription factor HNF4A binds to this enhancer region in an allele-specific manner, and knocking down *HNF4A* reduces the target gene *PRMT7*′s expression (Fig. 4). Sixth, public Hi-C data in human liver tissue from the 3D Genome Browser indicates that the region containing rs73613962 has a long-range interaction with the transcription start site (TSS) of *PRMT7* (Supplementary Fig. 22)^29^. These data strongly indicate that rs73613962 contributes to HCC in an enhancer-dependent manner.

A direct way to validate the interaction between an intronic enhancer and a TSS is to carry out a Chromosome Conformation Capture (3 C) experiment. However, suitable restriction enzyme cutting sites near these two potentially interacted regions could not be found. Nevertheless, the 3D Genome Browser provided by Wang et al. gives us an opportunity to validate such an interaction^29^, which provides online visualization of Hi-C data in human liver tissue. A high peak (reflecting a Hi-C rad value) between the TSS and rs73613962 is observed (Supplementary Fig. 22). In addition to the binding of HNF4A on the rs73613962-residing region, the promoter region of *PRMT7* also shows HNF4A enrichment, examined using HNF4A-ChIP-qPCR and ChIP-PCR (Supplementary Figs. 23 and 24), further supporting the physical interaction of this intronic enhancer with its host gene′s promoter.

Multiple lines of evidence supporting that HNF4A binds to the sequence around rs73613962 to act as an enhancer are provided in this study. In addition, HNF4A-ChIP-seq in Caco2 cells also supports such direct binding^30^. However, when investigating the sequence encompassing rs73613962, we find it does not contain the canonical binding motif of HNF4A. Transcription factor binding motifs are usually characterized using genome-scale technologies in vivo or in vitro, e.g. ChIP-seq and protein-binding microarrays, combined with statistical inference^31, 32^. The summarized motif is representative of all potential sequences, but it does not mean that all of the binding sequences should be exactly the same^31^. Actually, the known HNF4A-binding sequences CAAAGTCCA and AGTCCA occupy only 8% and 32% of the ChIP-seq peaks, respectively^33^. Therefore, we speculate that the sequence surrounding rs73613962 may be the additional target of HNF4A and deserves in-depth investigation.

PRMT7 belongs to the PRMT family, which is an important player of post-translational modification functioning on epigenetics and gene regulation^34^. The overexpression of *PRMT7* was reported to promote the progression of breast cancer^35–37^ and non-small-cell lung cancer^38^, but the role of PRMT7 in HCC has not yet been explored. Furthermore, no study has yet demonstrated whether there is any genetic variant within a gene regulatory region related to *PRMT7* influencing the expression or function of *PRMT7* as well as contributing to disease risk or progression. In this study, we implicate *PRMT7* as the target of the rs73613962-harboring enhancer, based on *cis*-eQTL analysis in GTEx liver tissue and TCGA LIHC (Fig. 3a, b). Both CRISPR-Cas9 and dCas9 assays provide further evidence that *PRMT7* expression is regulated by the rs73613962-residing enhancer region (Fig. 3c–i). Additionally, *PRMT7* expression is affected in accompany with the interference of the expression of transcription factor HNF4A bound to this enhancer-promoter interacting region (Fig. 4f). Subsequently, we demonstrate that the dynamic expression of *PRMT7* affects HCC pathogenesis through a series of experiments, with *PRMT7*-downregulated HCC cell lines having a decreased proliferation rate, and attenuated migration and invasion abilities (Fig. 5a–i). IHC staining and TCGA LIHC also show that PRMT7 is highly expressed in HCC (Fig. 5j–l), further suggesting its critical function in HCC tumorigenesis, acting as the target gene of the susceptibility locus rs73613962.

Mechanistically, we investigate that the p53 signaling pathway may be involved in the PRMT7-mediated HCC cell malignant phenotypes. Classified as type III arginine methyltransferase, PRMT7 catalyzes the MMA formation of substrates, of which histone is the known substrate^39–41^. Additionally, it has been reported that the level of H4R3me2s, the repressive histone mark, was reduced when *PRMT7* was deficient^42, 43^. In our study, we firstly find the signal of H4R3me2s is significantly decreased when compared *PRMT7*-downregulated samples to the control (Fig. 6d). Further, we investigate that the genes enriched in the p53 signaling pathway contain CAGCTG motif of H4R3me2s modification region (Fig. 6e–h). These genes′ expression is upregulated in *PRMT7*-downregulated cells implicated by RNA-seq data and verified by qRT-PCR (Fig. 6c). For example, we confirm the promoter region of *CDKN1A*, an important component of the p53 signaling pathway, does have H4R3me2s modification which has been reported that its expression can be regulated by PRMT7 through affecting DNA methylation and H4R3me2s modification of its promoter region^44^. Additionally, we verify that the RNA level of *CDKN1A* and the protein level of its encoding protein, p21, are significantly increased in *PRMT7*-depleted cells (Fig. 6c and Supplementary Fig. 25). These lines of evidence together imply that PRMT7 may promote HCC cell malignant phenotypes mediated by regulation on H4R3me2s modification of genes enriched in the p53 signaling pathways.

However, it is also possible that some non-chromatin factors might be involved in PRMT7-inducing carcinogenesis because a few nonhistone interactors or substrates of PRMT7 have been identified^45–47^. For example, some differentially expressed genes are categorized into Ribosome and Splicesome in our study, which has been identified to be the enriched function classification of the methylated proteins^47^. The possibly regulated RNA binding proteins by PRMT7 are warranted to be further investigated in the future.

Conclusively, a HCC-susceptible SNP rs73613962 at an intronic enhancer region of *PRMT7* is identified, and its causal role in cancer-related phenotypes is demonstrated. Transcription factor HNF4A is found to bind to the rs73613962-containing enhancer region to promote the expression of the host gene *PRMT7*, which ultimately contributes to HCC pathogenesis. This work may shed a light on the pathogenesis of HCC and has potential implications on HCC prevention/treatment.

## Methods

The protocol of the study was approved by the Ethical Committee of Nanfang Hospital, Southern Medical University (Guangzhou, China), the Ethics Committee of Qidong Liver Cancer Institute (Qidong, China), the Ethics Committee of the Second Military Medical University (Shanghai, China), the Ethical Committee of Soochow University (Suzhou, China), the Ethical Committees of Beijing Ditan Hospital and Beijing You’an Hospital (Beijing, China), and the Ethic Committee of Youjiang Medical College for Nationalities (Baise, China).

### Study subjects and genotyping

We conducted a three-stage study, which included 4898 HCC cases and 7060 controls, to screen and validate HCC risk-associated genetic variant(s) residing in enhancer regions throughout the whole genome. The demographic characteristics of all the study subjects included in the three stages are summarized in Supplementary Table 1. In the discovery stage, a total of 2514 study subjects who were all chronic HBV carriers consisting of 1161 HCC cases and 1353 non-HCC controls from East China (Qidong, Jiangsu province) were included. All the cases and controls were recruited by Qidong Liver Cancer Institute in Qidong County, Jiangsu Province, China, during the period from May 2006 to December 2012, which was approved by the Ethics Committee of Qidong Liver Cancer Institute. Genomic DNA samples from all the subjects were genotyped for 731,442 SNPs across the whole genome using Illumina Human OmniExpress BeadChips, which have been previously described in detail^5^. After the screening process shown in Fig. 1a, six SNPs were screened out from the discovery stage for validation in the following replication stages. At replication stage 1, a total of 1297 chronic HBV carriers including 576 HCC cases and 721 non-HCC controls were enrolled from East China (Shanghai). All the subjects were recruited from the affiliated hospitals of the Second Military Medical University, Shanghai, China, from October 2009 to September 2011, which was approved by the Ethics Committee of the Second Military Medical University. Genomic DNA samples extracted from all the subjects at replication stage 1 were genotyped for the six selected SNPs, using the improved multiple ligase detection reaction (iMLDR) method (Genesky Biotechnologies Inc., China). At replication stage 2, a total of 8147 chronic HBV carriers, including three independent subject sets (the replication stage 2a: 1942 HCC cases and 2812 non-HCC controls; the replication stage 2b: 393 HCC cases and 1314 non-HCC controls; the replication stage 2c: 826 HCC cases and 860 non-HCC controls), were recruited from East China (Shanghai and Jiangsu province), North China (Beijing and Shandong province), and South China (Guangxi province), respectively. The study subjects recruited in replication stage 2a were diagnosed, hospitalized, and treated in the affiliated hospitals of Soochow University or the Suzhou Municipal Hospital from 2007 to 2010, which was approved by the Ethical Committee of Soochow University. The subjects in replication stage 2b were recruited from Beijing Ditan Hospital and Beijing You’an Hospital during the period of November 2001 to August 2004, which was approved by the Ethical Committees of Beijing Ditan Hospital and Beijing You’an Hospital. The subjects in the replication stage 2c were enrolled by Youjiang Medical College for Nationalities during January 2004 and December 2010, which was approved by the Ethic Committee of Youjiang Medical College for Nationalities. All the subjects at replication stages 2a, 2b, and 2c were genotyped for rs73613962, which was validated at replication stage 1 using the TaqMan assay platform (ABI 7900HT System, Applied Biosystems).

The definition of chronic HBV carriers and HCC patients, as well as the inclusion and exclusion criteria for the recruitment of study subjects, were described in our previous study^5, 48^. Briefly, chronic HBV carriers were defined as positive for both hepatitis B surface antigen and antibody IgG to hepatitis B core antigen for at least 6 months. The diagnosis of HCC was based on (1) positive findings on cytological or pathological examinations, and/or (2) positive images on angiogram, ultrasonography, computed tomography, and/or magnetic resonance imaging, combined with an alpha-fetoprotein level ≥400 ng/ml. All HCC patients were confirmed to not have other cancers by initial screening. All the controls had no cirrhosis, HCC, or other cancers. All subjects were negative for antibodies to HCV, or human immunodeficiency virus, and had no other types of liver disease, including autoimmune hepatitis, toxic hepatitis, and primary biliary cirrhosis.

Signed informed consent was obtained from all study subjects before their participation in the study. The study was conducted in accordance with the Declaration of Helsinki principles and compliant with the “Guidance of the Ministry of Science and Technology (MOST) for the Review and Approval of Human Genetic Resources”.

### Culture of cell lines

The QGY-7703, HepG2, SMMC-7721, HepAD38, SNU398, and 293 T cell lines were originally purchased from the Cell Bank of the Chinese Academy of Sciences (Shanghai, China). QGY-7703, HepG2, SMMC-7721, HepAD38, and 293 T cell lines were cultured in Dulbecco′s modified Eagle′s medium (DMEM). SNU398 cell line was cultured in 1640 medium. The medium was supplemented with 10% fetal bovine serum (FBS). All cells were cultured at 37 °C in an incubator containing 5% CO_2_.

### Dual-luciferase report assay

First, the DNA sequences surrounding rs73613962 (harboring the non-risk allele T) were cloned into the pGL4.23 luciferase report vector. The fragments containing the risk allele G were obtained using the PCR-based site-directed mutagenesis method. The two vectors were then verified using Sanger sequencing and did not contain any other variants. Then, the two constructs and empty vector were separately transfected into cells using lipofectamine 3000 (Thermo Fisher) in 24-well plates (four-well per each vector). The pRLTK *Renilla* Luciferase vector acted as the internal control and was co-transfected into cells with the above luciferase report vector. After transfection for 24 h, the *Firefly* and *Renilla* luciferase activities were measured one by one according to the manufacturer′s instruction (Promega). The final *Firefly* luciferase activity was normalized to the *Renilla* luciferase value. The primers used are listed in Supplementary Data 2.

### Editing enhancer region by CRISPR-Cas9

Two gRNA oligos targeting the upstream and downstream of the rs73613962-harboring regions were designed using online CRISPR Design (http://crispr.mit.edu/), and then were inserted into the plasmid pBC2-Cas9 at the SalI and AfII restriction site. The plasmid (pBC2-Cas9-gRNA) was transfected into cells by lipofectamine 3000 (Thermo Fisher) in a six-well plate. Twenty-four hours after transfection, cells were screened using 400 μg/ml hygromycin for 2 days, then the surviving cells were trypsinized, diluted, and seeded into 96-well plates for single-cell clone selection. The gRNA oligos are listed in Supplementary Data 2.

### Enhancer repression and activation by dCas9

Three guide RNA (gRNA) oligos surrounding rs73613962 were inserted into the pBC2-dCas9-KRAB (CRISPRi) or pBC2-dCas9-VP48 (CRISPRa) vectors at the SalI and AfII restriction site. The dCAS9-gRNA-expressed construct and control vector were separately co-transfected with the pBase vector into cells by lipofectamine 3000 (Thermo Fisher) in six-well plates. Twenty-four hours post-transfection, positive cells were selected by replacing DMEM medium containing hygromycin at a final concentration of 400 μg/ml for a week. The gRNA oligos are listed in Supplementary Data 2.

### Quantitative real-time polymerase chain reaction

Total RNA and protein were extracted using TRIzol reagent (Sigma) according to the manufacturer′s instruction. For cDNA synthesis, 1 μg total RNA was reverse-transcribed into cDNA using the FastQuant RT Kit (TIANGEN). Then quantitative PCR was performed using 2 × SYBR mix (Vazyme), and the reaction was run on the Bio-Rad CFX manager machine. The sequence information of all primers is listed in Supplementary Data 2.

### Electrophoretic mobility shift assay

DNA oligonucleotides designed for EMSA were centered around rs73613962 with alleles T or G. The two pairs of oligos were labeled with biotin at both the 5′ ends, and the sequences are listed in Table S4. The procedure of EMSA was performed following the manufacturer′s protocol (LightShift ® Chemiluminescent EMSA Kit, Thermo Fisher). In our study, 25 fmol labeled oligos were incubated with nuclear lysate. For competition and blocking experiments, the reaction solution with nuclear lysate was preincubated with unlabeled oligos or anti-HNF4A antibody (Abcam, cat. no. ab181604) for 10 min (min) on ice. The reactions were then run on 6% polyacrylamide gel in 0.5 × TBE, then transferred and crosslinked to nylon membrane. After the membrane was blocked with streptavidin-horseradish peroxidase (HRP) conjugate and washed with the washing buffer, labeled DNA was detected by chemiluminescence. Visualization was finally carried out using the Tanon Chemiluminescent Imaging System.

### Chromatin immunoprecipitation assay

The ChIP assay was performed using the SimpleChIP^®^ Plus Sonication Chromatin IP Kit purchased from CST (cat. no. 56383). Briefly, cells were first crosslinked with 1% formaldehyde in 10 cm culture dishes for 15 min. After being quenched with glycine solution, cells were washed and scraped to harvest cell pellets. Then, the nucleic fraction was isolated from the cell lysate by resuspending separately with the ChIP Sonication Cell Lysis Buffer and ChIP Sonication Nuclear Lysis Buffer. After being fragmented via Bioruptor sonication, the chromatin was incubated with anti-HNF4A antibody (2 μg per reaction, Abcam, cat. no. ab181604) or H4R3me2s (5 μg per reaction, Active motif, cat.no. 61187) or IgG for 4 h at 4 °C with rotation. Then, the ChIP-Grade Protein G beads were added to each immunoprecipitation reaction and incubated for another 2 h at 4 °C with rotation. The captured protein-DNA was then de-crosslinked using 5 M NaCl and Proteinase K for 2 h at 65 °C. Finally, DNA was purified with spin columns according to the manufacturer′s protocol. The enriched DNA was then subjected to downstream assays (ChIP-qPCR and ChIP-PCR). All primer sequences used for ChIP-qPCR/PCR are listed in Supplementary Data 2.

### Transient transfection

Cells were seeded in 12-well plates for 50–60% confluence 1 day in advance and then were transfected with siRNAs targeting *HNF4A* or *PRMT7* and the control siRNA using lipofectamine 2000 (Thermo Fisher) according to the manufacturer′s instruction on the second day. Cells were harvested for RNA extraction 48 h later. The siRNAs used in this study were synthesized and purchased from GenePharma (Shanghai, China) and are listed in Supplementary Data 2.

### Lentivirus transduction

*PRMT7* shRNA plasmids were constructed by annealing the DNA oligonucleotides and then individually cloning them into the pLKO.1 plasmid at the EcoRI and AgeI restriction enzyme sites. The *PRMT7*-overexpressed vectors were first constructed to obtain the complete coding sequence by PCR amplification from human cell cDNA, and then inserted into the pcDH_EF1_MCS_T2A_Puro vector, respectively. The plasmids obtained above were finally validated by Sanger sequencing. The DNA oligonucleotides and primer sequences are listed in Supplementary Data 2. Then, the expression vector or the control vector plus VSVG and gag/pol encoding plasmids were transfected into the 293 T cells, respectively. 24 h and 48 h later, the virus supernatant was harvested to infect target cells, respectively. Finally, the medium was replaced with a fresh DMEM medium supplemented with puromycin (the final concentration was 2.5 μg/ml) to screen stably transformed cells after the second infection.

### Western blot

The primary antibodies for detecting PRMT7 (dilution: 1:1000, Abcam, cat. no. ab179822), H4R3me2s (dilution: 1:500, Active motif, cat.no. 61187), H4 (dilution: 1:500, Abcam, cat. no. ab177840), p21 (dilution: 1:1000, CST, cat. no. 2947 S), and GAPDH (dilution: 1:1000, CST, cat. no. 2118 S) were diluted using 5% non-fat milk buffer and were incubated with the membrane for 2 h at room temperature. Then, the membrane was incubated with the second antibody (dilution: 1:5000, anti-Rabbit IgG, HRP conjugate, YEASON) for 1 h at room temperature. The membrane was washed three times with TBST after each incubation and visualization was carried out using the Tanon Chemiluminescent Imaging System.

### Cell proliferation assay and cell cycle analysis

Cells were diluted and seeded into a 96-well plate, with 2000 cells per well and three replicates for each time point. Then, the CCK-8 reagent was added to each well at 24, 48, 72, and 96 h post-seeding, according to the manufacturer′s protocol. After incubation for 2 h at 37 °C, the optical densities at 450 nm and 600 nm were separately measured for individual wells using a microplate reader (Tecan i-control).

For cell cycle analysis, cells were centrifuged at 500 × *g* for 4 min. Then, the cell pellets were washed once using 1 × PBS and resuspended using 1 × PBS containing 0.03% Triton X-100 and 50 μg/ml propidium iodide. After incubation at room temperature for 10 min, the cell cycle assay was performed on the BD Flow Cytometer.

### Colony formation assay

Cells were seeded into a six-well plate (2 × 10^3^ cells per well). Fresh DMEM was resupplied every 3 days, and cells were cultured under this condition for 2 weeks. Next, cells were fixed using 4% paraformaldehyde and incubated for 15 min at room temperature. Then, 0.2% crystal violet staining solution was added to each well and incubated with cells for another 30 min. After being washed three times with 1 × PBS, finally, the plates were dried and photographed.

### Cell migration and invasion

First, cells were individually trypsinized and resuspended in FBS-free medium. The cell suspension was aspirated into the upper layer of the transwell chamber (3 × 10^4^ cells per well), with FBS-containing medium in the lower well. The medium of the upper layer was removed 48 h later, and the cells were fixed with 4% paraformaldehyde for 15 min. After being washed two times with 1 × PBS, the cells were incubated with 0.2% crystal violet staining solution for an additional 30 min. Then, the wells were washed again with 1 × PBS at least four times, and the cells of the upper layer were removed gently using a cotton swab. Finally, the results were observed under a microscope.

### Nude mice tumorigenicity assay

BALB/c Nude mice (female, 4 weeks old) purchased from the Beijing Vital River Laboratory Animal Technology Co., Ltd. were randomly divided into three groups (nine mice per group). These mice were housed in the specific pathogen-free (SPF) environment at a constant temperature (26–28 °C) and a relatively constant humidity (40–60%), with 10 h light/14 h dark cycle. 1 × 10^7^ QGY-7703 control cells (Control) and *PRMT7*-knockdown cells (*PRMT7*-sh1 or *PRMT7*-sh2) were individually inoculated into the right flank of the nude mice. Seven days after the injection, the tumor size, including the tumor length and width, was measured twice a week. At the end of 21 days, the nude mice were sacrificed and dissected, and both the tumor size and tumor weight were measured. The tumor volume was calculated by π/6 × length × width^2^. All animal experiments were approved by the Ethical Committee for Experimental Animal Care of Nanfang Hospital, Southern Medical University (Guangzhou, China), and compliant with the rules on an experimental mouse for tumor research that the tumor weight of mice should not exceed 10% of the bodyweight of mice, and the average tumor diameter should not exceed 20 mm.

### Immunohistochemistry assay

Sections were deparaffined in xylene and rehydrated in alcohol. Endogenous peroxidase activity was blocked using 3% H_2_O_2_ for 10 min. Antigen retrieval was achieved by heating twice using a microwave in a citrate acid buffer (pH 6.0), and sections were blocked in 3% goat serum (Boster, China) at 37 °C for 15 min. Slices were incubated with primary antibody PRMT7 (dilution: 1:100, Abcam, cat. no. ab181214) at 4 °C overnight, followed by three washes in 1 × PBS, incubation with the corresponding biotin-labeled secondary antibodies for 30 min, three more washes in 1 × PBS, and incubation with a streptavidin-biotin complex for 25 min. Immunostaining was achieved using 3,3′-diaminobenzidine tetrahydrochloride. All slides were counterstained with hematoxylin.

### RNA-sequencing and data analysis

The RNA-seq library was constructed according to the KAPA HyperPrep Kits′ instruction (Roche, KK8504). Briefly, polyA+ RNA was first captured by two rounds of selection with oligo(dT) beads. The first-strand DNA synthesis was immediately performed following the fragmentation of enriched poly A + RNA. Then, the second-strand DNA synthesis, dA-tail adding of 3′ ends, and Y-shape linker ligation was performed one by one according to the protocol. After three rounds of purification with pure beads, a PCR of 14 cycles was carried out. The final libraries were sequenced using the Illumina HiSeq platform.

For the RNA-seq data analysis, the FastQC software was first used for quality control of the RNA-seq data, and nucleotides with sequencing quality smaller than 20 were trimmed off. After the RNA-seq reads were mapped to the hg19 genome using the tophat2 (v2.1.1) software, the mRNA abundance and gene expression change were evaluated using GFOLD (v1.1.4) software. The functional enrichment of the differentially expressed genes was finally analyzed through the DAVID (v6.8), of which the KEGG PATHWAY Database was selected.

### Statistical and bioinformatical analyses

We did association analyses using PLINK (v1.07) for over 4.8 million SNPs across the whole genome at the discovery stage, for six SNPs (selected from the discovery stage) at replication stage 1, and for rs73613962 (validated by the replication stage 1) at replication stage 2. At the discovery stage, in order to increase the genome coverage, we carried out an imputation analysis on the basis of the genotyped SNPs in the pre-existing GWAS (568,280 SNPs in 1161 cases and 1353 controls retained for analysis after a standard process of quality control, which has been described previously^5^) and haplotype data of Han Chinese in Beijing, China (CHB) from the 1000 Genome Project by using the Michigan imputation server (https://imputationserver.sph.umich.edu/). SNPs with a minor allele frequency (MAF) less than 0.05 or significantly deviated from the Hardy-Weinberg equilibrium (HWE, *P* < 0.0001) were removed from further analyses. In all three stages, logistic regression was used to evaluate the association of each SNP with HCC risk under an additive model and adjusted for gender and age. A meta-analysis of the association results of the three stages was performed using the fixed-effects model (Mantel-Haenszel model).

The association of *PRMT7* expression with clinical outcome of HCC patients was performed in LIHC subjects of TCGA Program. Survival curves were estimated according to the Kaplan–Meier method, and the statistical differences in the survival curves of different subgroups of subjects were analyzed using the log-rank test.

For *cis*-eQTL analysis, linear regression was used to evaluate the association of the rs73613962 genotype with the expression of *PRMT7* in both the data of liver tissues in the GTEx Project and the data of LIHC subjects of TCGA Program without or with stratification according to the status of HBV infection or ethnicity (Asians vs. Europeans) when it is appropriate. For co-expression analysis, Spearman′s rank correlation coefficient was used to evaluate the correlation of the expression of *PRMT7* and the expression of *HNF4A* in both the GTEx liver tissue data and TCGA-LIHC data.

### Reporting summary

Further information on research design is available in the Nature Research Reporting Summary linked to this article.

## Supplementary information

Supplementary Information

Description of Additional Supplementary Files

Supplementary Data 1

Supplementary Data 2

Supplementary Data 3

Reporting Summary

## Data Availability

The summary statistics for the top 10,000 SNPs in association with HCC risk at the discovery stage are provided in Supplementary Data 3. The raw genotype data of HCC and CHB patients, which are considered as genetic information of Chinese population and restricted for being publicly available by the China’s Ministry of Science and Technology (MOST), will be allowed to obtain for all the individuals engaged in academic research by contacting the corresponding author. Before we send the data, a data confidentiality contract needs to be signed to ensure that the use of the data will meet the requirements of MOST and the data will not be leaked to a third party. Once the contract is signed, we will share the data immediately. The RNA-seq data of QGY-7703 cell line before and after PRMT7 knockdown (QGY-7703_control, QGY-7703_PRMT7_sh1, QGY-7703_PRMT7_sh2) have been deposited in the GEO database at NCBI under accession number GSE167432. The data used for *cis*-eQTL in this study are publicly available from the GTEx portal and TCGA portal. GTEx data can be accessed through the GTEx Portal (https://www.gtexportal.org/home)^16^, and TCGA data can be accessed through the Genomic Data Commons Data Portal (https://portal.gdc.cancer.gov). KEGG PATHWAY Database can be accessed through KEGG Database (https://www.kegg.jp/kegg/). The publicly available ChIP-seq data of three kinds of histone modification (H3K4me3, H3K4me1, and H3K27ac) and transcription factor, and the data of DNase I hypersensitive site used in this study are derived from ENCODE datasets accessed through ENCODE (https://www.encodeproject.org/). The publicly available HNF4A-ChIP-seq data in Caco2 used in this study can be accessed in the GEO database at NCBI under accession number GSE23436^30^. The publicly available H4R3me2s ChIP-seq data in mouse embryonic stem cells used in this study can be accessed in the GEO database at NCBI under accession number GSE37604^24^. Hi-C data in liver can be accessed in the GEO database at NCBI under accession number GSE58752^49^. Source data are provided with this paper.

## Source data

Source Data
