# Supplementary material for: An enhancer variant at 16q22.1 predisposes to hepatocellular carcinoma via regulating PRMT7 expression
Source: Nat Commun. 2022 Mar 9;13:1232. doi: 10.1038/s41467-022-28861-0 (PMC8907293; doi:10.1038/s41467-022-28861-0)

# Supplementary Information

## An enhancer variant at 16q22.1 predisposes to hepatocellular carcinoma via regulating PRMT7 expression

Ting Shen[1,2†], Ting Ni[3†], Jiaxuan Chen[1†], Haitao Chen[1,4†], Xiaopin Ma[3†], Guangwen Cao[5†], Tianzhi Wu[6], Haisheng Xie[1], Bin Zhou[1], Gang Wei[3], Hexige Saiyin[3], Suqin Shen[3], Peng Yu[3], Qianyi Xiao[7], Hui Liu[8], Yuzheng Gao[9], Xidai Long[10], Jianhua Yin[5], Yanfang Guo[6], Jiaxue Wu[3], Gong-Hong Wei[11,12], Jinlin Hou[1], De-Ke Jiang[1]*

[1]State Key Laboratory of Organ Failure Research, Guangdong Key Laboratory of Viral Hepatitis Research, Guangdong Institute of Liver Diseases, Department of Infectious Diseases and Hepatology Unit, Nanfang Hospital, Southern Medical University, Guangzhou 510515, China.
[2]School of Life Sciences, Central South University, Changsha 510006, China.
[3]State Key Laboratory of Genetic Engineering, Collaborative Innovation Center for Genetics and Development, Human Phenome Institute, School of Life Sciences, Fudan University, Shanghai 200438, China.
[4]School of Public Health (Shenzhen), Sun Yat-sen University, Shenzhen 528406, China.
[5]Department of Epidemiology, Naval Medical University, Shanghai 200433, China.
[6]Institute of Bioinformatics, School of Basic Medical Science, Southern Medical University, Guangzhou, Guangdong 510515, China.
[7]School of Public Health, Fudan University, Shanghai 200032, China.
[8]School of Basic Medical Sciences; The Sixth Affiliated Hospital of Guangzhou Medical University, Qingyuan People's hospital, Guangzhou Medical University, Guangzhou 510182, China.
[9]Department of Forensic Medicine, Medical College of Soochow University, Suzhou, Jiangsu Province 215123, China.
[10]Department of Pathology, Youjiang Medical College for Nationalities, Baise, Guangxi Province 533000, China.
[11]Biocenter Oulu, Faculty of Biochemistry and Molecular Medicine, University of Oulu, Oulu 90014, Finland.
[12]School of Basic Medical Sciences, Fudan University, Shanghai 200032, China.
[†]These authors contributed equally to this work: Ting Shen, Ting Ni, Jiaxuan Chen, Haitao Chen, Xiaopin Ma, Guangwen Cao.

***Corresponding author**
Prof. De-Ke Jiang,
Department of Infectious Diseases and Hepatology Unit, Nanfang Hospital, Southern Medical University, Guangzhou 510515, China.
Telephone: +86-20-62786533
Email: dekejiang17@smu.edu.cn

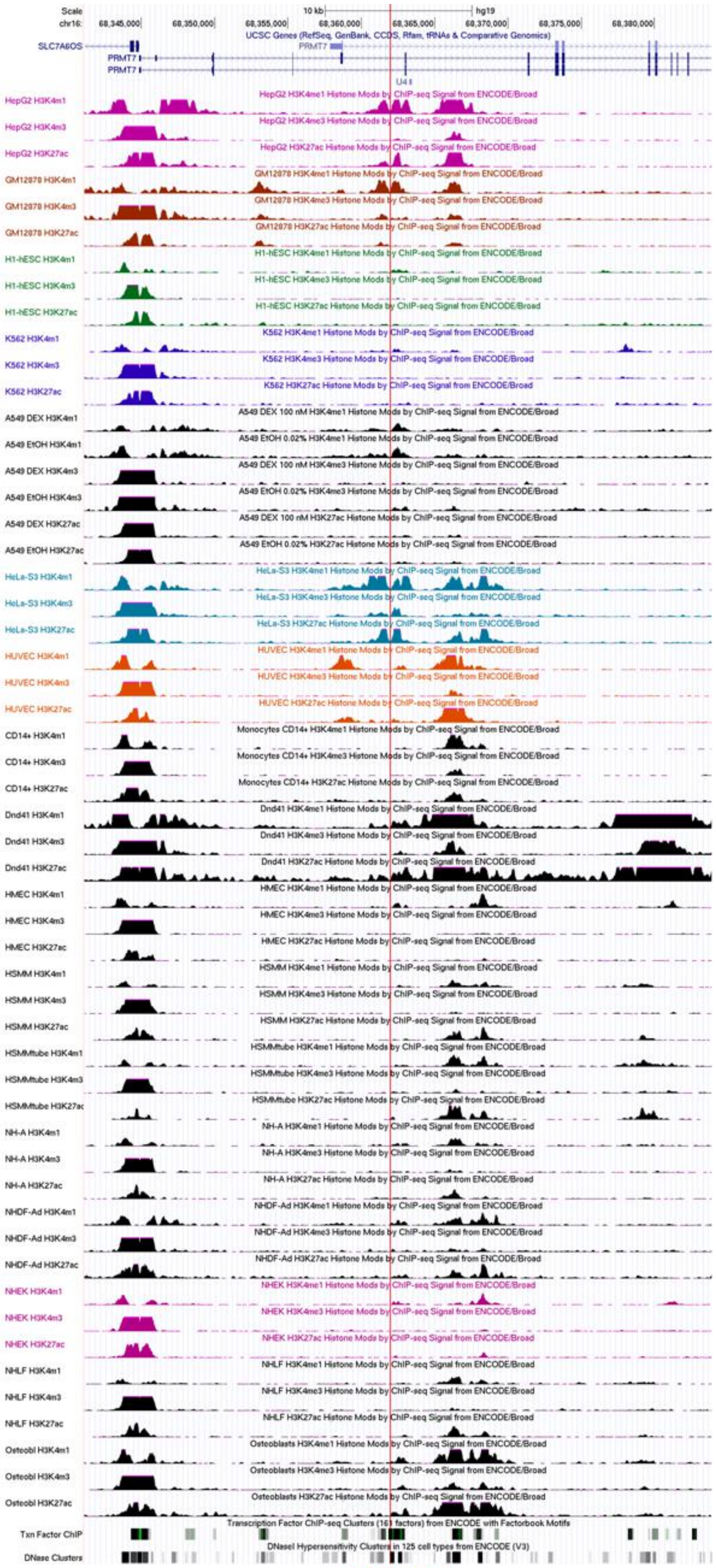
Scale
chr16:
10 kb
hg19
UCSC Genes (RefSeq, GenBank, CCDS, Rfam, tRNAs & Comparative Genomics)
SLC7A6OS
PRMT7
U4
Transcription Factor ChIP-seq Clusters (161 factors) from ENCODE with Factorbook Motifs
DNaseI Hypersensitivity Clusters in 125 cell types from ENCODE (V3)
Txn Factor ChIP
DNase Clusters
rs73613962

**Supplementary Figure 1 The rs73613962-containing region is a strong transcriptional regulatory element in HepG2.** Overview of the H3K4me1, H3K27ac, and H3K4me3 chromatin modifications, the binding sites of transcription factors (Txn Factor ChIP), and DNase cluster distribution in the region surrounding rs73613962 of different cell lines from UCSC genome browser. Rs73613962 is indicated by the red vertical line.

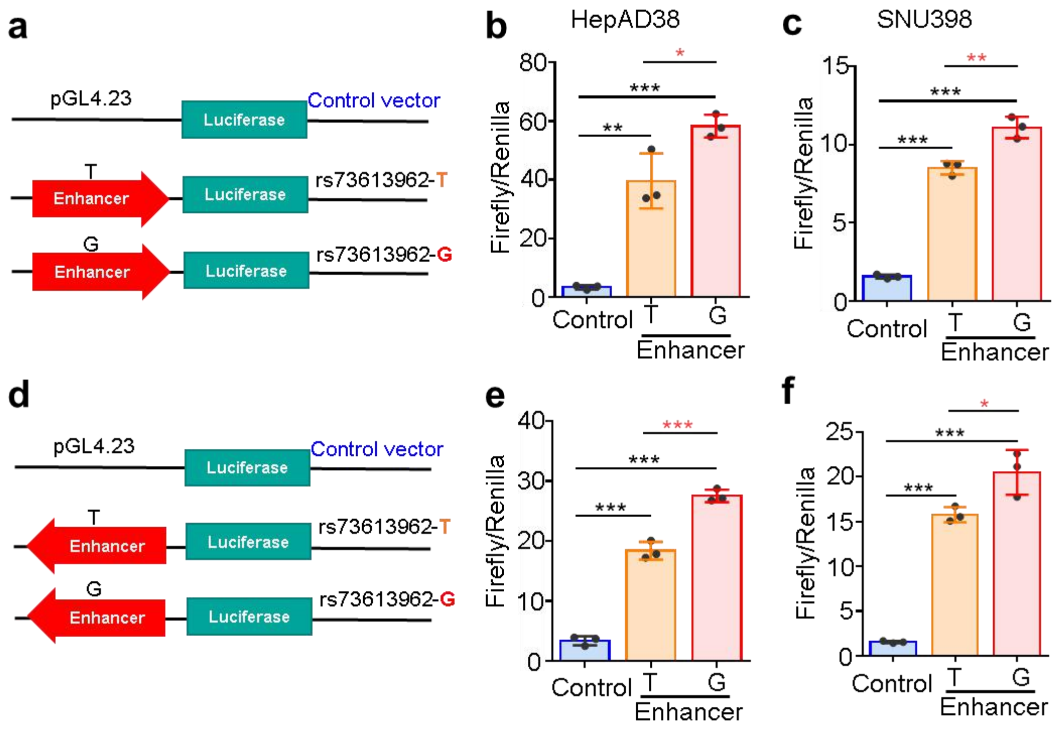


**Supplementary Figure 2 Dual luciferase reporter assay of enhancer activity.** Luciferase activity is individually examined when rs73613962-centred region is inserted in luciferase vector at forward (**a-c**) or reverse orientation (**d-f**). The assays are performed in HepAD38 (**b**, $P$ = 0.0026 and $P$ < 0.0001 in Enhancer-T or Enhancer-G compared to Control, and $P$ = 0.0330 in Enhancer-G compared to Enhancer-T; **e**, $P$ < 0.0001 in Enhancer-T or Enhancer-G compared to Control, and $P$ = 0.0010 in Enhancer-G compared to Enhancer-T) SNU398 (**c**, $P$ < 0.0001 in Enhancer-T or Enhancer-G compared to Control, and $P$ = 0.0053 in Enhancer-G compared to Enhancer-T; **f**, $P$ < 0.0001 and $P$ = 0.0002 in Enhancer-T or Enhancer-G compared to Control, and $P$ = 0.0355 in Enhancer-G compared to Enhancer-T), respectively. Values are expressed as the mean ± SD, $n$ = 3 in **b**, **c**, **e**, and **f**. *, **, *** mean $P$ value less than 0.05, 0.01, and 0.001, respectively (two-sided student' $t$-test).

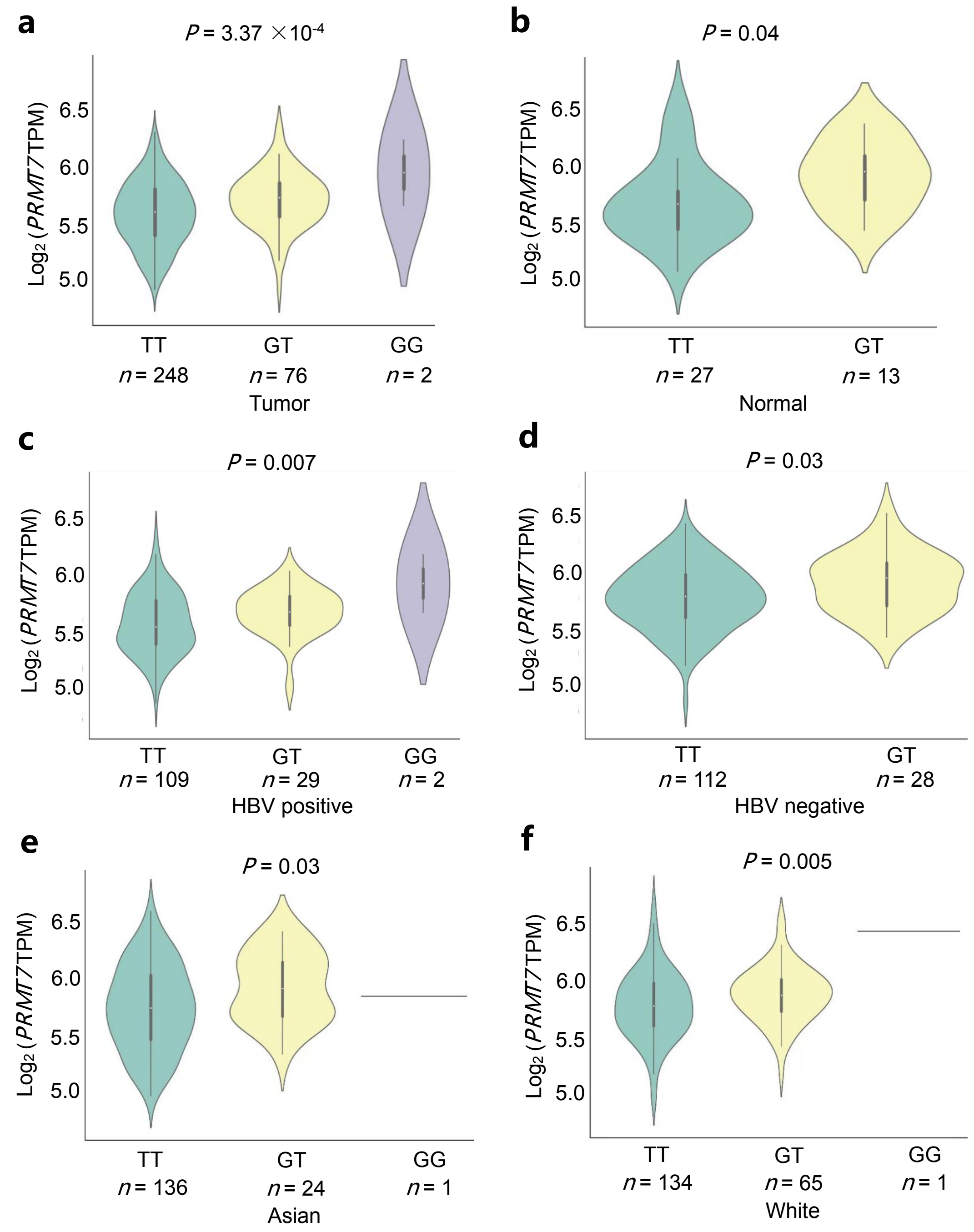


**Supplementary Figure 3 Rs73613962 is associated with the expression of *PRMT7* in liver hepatocellular carcinoma (LIHC) of TCGA.** The association between rs73613962 genotypes and *PRMT7* expression in the tumor samples (**a**, $n$ = 326), in the normal samples (**b**, $n$ = 40), in the HBV-positive samples (**c**, $n$ = 140), in the HBV-negative samples (**d**, $n$ = 140), in Asian samples (**e**, $n$ = 161), and in White (European) samples (**f**, $n$ = 200) of LIHC through linear regression analysis. *PRMT7* expression value is $Log_2$ transformed. TPM, transcripts per million. The center white dot, the black limit, and the whisker in each violin plot represent the median, the first to third quartile, and the 95% confidence interval, respectively.

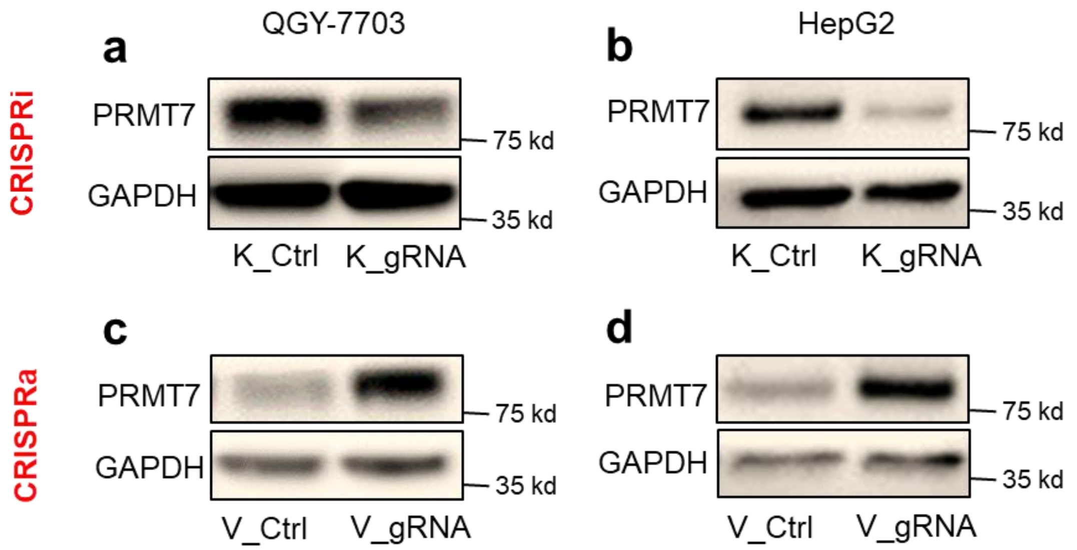


**Supplementary Figure 4 PRMT7 protein level is decreased in CRISPRi and increased in CRISPRa.** Western blot detection of PRMT7 protein abundance in CRISPRi (**a**, **b**) and CRISPRa (**c**, **d**) assays, respectively. The two experiments were individually performed in the QGY-7703 (**a**, **c**) and HepG2 (**b**, **d**) cell lines. The experiments are replicated two times at least and similar results are observed.

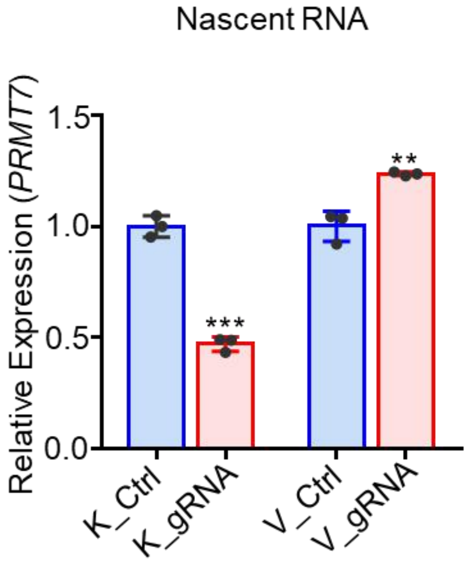


**Supplementary Figure 5 The rs73613962-harboring region regulates *PRMT7* expression on transcriptional level.** The nascent RNA assay was performed with the BrU method[1]. Briefly, BrU, a nucleotide analog, was added into the medium and incubated with cells of CRISPRi and CRISPRa assays for 1 h at 37°C, respectively. Therefore, BrU would be introduced into the newly transcribed RNA. Then the BrU-contained RNAs were enriched from the total RNA through incubation with anti-BrU antibody (BD Pharmingen). Finally, BrU-RNAs were reversely transcribed to cDNA, and the nascent *PRMT7* transcripts were then quantified by qPCR. The above picture shows the relative nascent PRMT7 expression detected by qPCR in CRISPRi and CRISPRa assays, respectively. $P < 0.0001$ and $P = 0.0042$ in K_gRNA compared to K_Ctrl and in V_gRNA compared to V_Ctrl, respectively. Values are expressed as the mean ± SD, $n = 3$. ** and *** mean $P$ value less than 0.01 and 0.001, respectively (two-sided student' $t$-test).

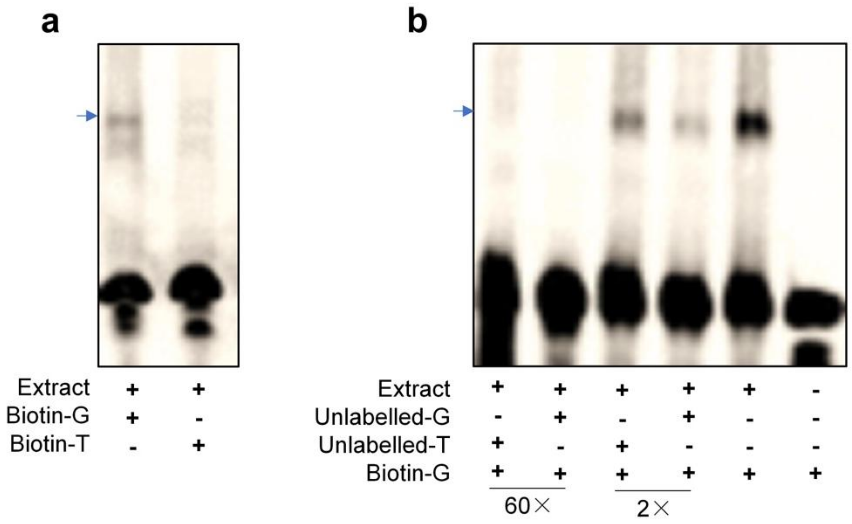


**Supplementary Figure 6 The results of EMSA in the QGY-7703 cell line. a** EMSA for biotin-labeled oligos containing the allele G (Biotin-G) or allele T (Biotin-T) of rs73613962. **b** The result of competitive EMSA. The binding preference of protein to DNA oligos was demonstrated by adding unlabeled-G or unlabeled-T into the reaction. The light blue arrows indicate the protein-biotin-G-oligo complexes. This experiment is repeated two times at least and similar results are observed.

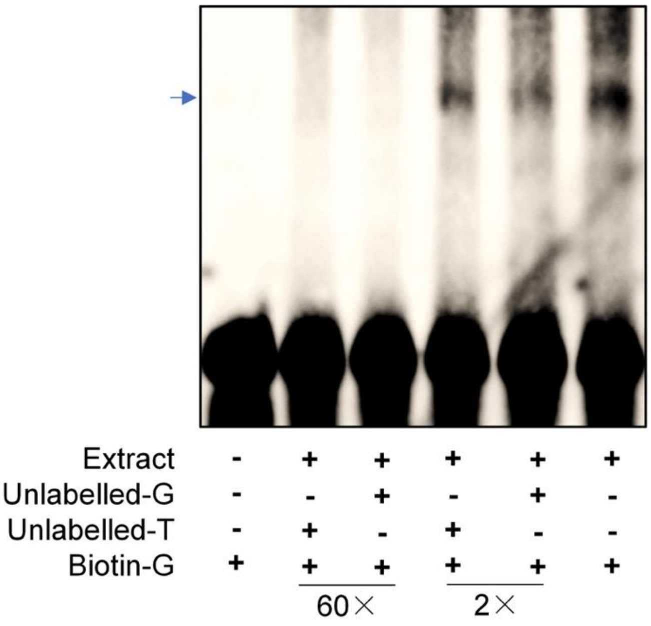


**Supplementary Figure 7 The result of competitive EMSA in the HepG2 cell line.** In this competitive EMSA, ~5 μg of nuclear extracts from the HepG2 cell line were used. The binding preference of protein to DNA oligos was demonstrated by adding unlabeled-G or unlabeled-T into the reaction. The light blue arrow indicates the protein-biotin-G-oligo complexes. This experiment is replicated two times at least and similar results are observed.

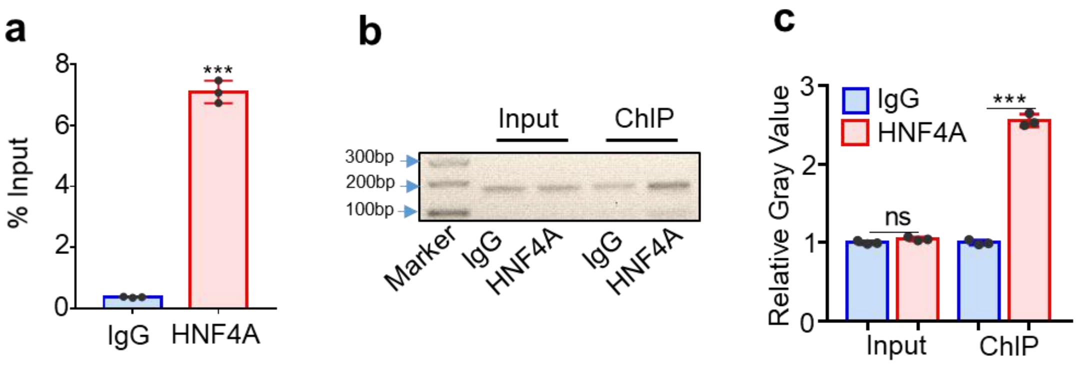


**Supplementary Figure 8 ChIP-qPCR and ChIP-PCR assays for the binding of transcription factor HNF4A on the rs73613962-containing region in the HepG2 cell line. a** qPCR detection for the enrichment of the rs73613962-containing region upon anti-HNF4A ChIP and IgG in the HepG2 cell line. $P < 0.0001$ in HNF4A compared to IgG. **b** The agarose gel electrophoresis of PCR products for Input and ChIP, respectively. The light blue arrows indicate the marker bands. **c** Relative gray value of each PCR band in (**b**) analyzed by ImageJ v1.4.3.67. $P = 0.0656$ and $P <$ 0.0001 in HNF4A compared to IgG in Input and ChIP, respectively. Values are expressed as the mean ± SD, $n = 3$ in **a** and **c**. 'ns' means not significant; *** means $P$ value less than 0.001 (two-sided student' $t$-test).

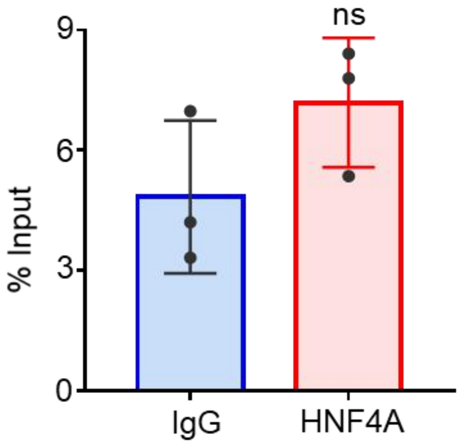


**Supplementary Figure 9 ChIP-qPCR assay for the binding of transcription factor HNF4A on the downstream region of rs73613962.** qPCR detection for the enrichment of the downstream region of rs73613962 upon anti-HNF4A ChIP and IgG. $P$ = 0.1779 in HNF4A compared to IgG. Values are expressed as the mean ± SD, $n$ = 3. ‘ns’ means not significant, i.e., $P$ value > 0.05 (two-sided student’ $t$-test).

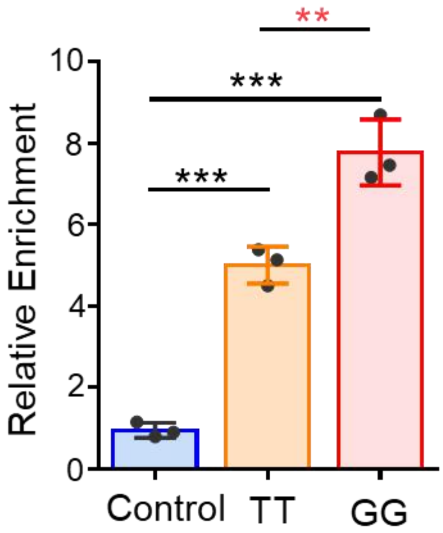


**Supplementary Figure 10 Relatively higher enrichment of HNF4A on the G allele of rs73613962.** We firstly transfected the vector having the insertion of G or T-centered sequence into the cells, and then conducted the HNF4A ChIP. *P* = 0.0001 and *P* = 0.0001 in TT or GG compared to Control, and *P* = 0.0068 in GG compared to TT. Values are expressed as the mean ± SD, *n* = 3. ** and *** mean *P* value less than 0.01 and 0.001, respectively (two-sided student' *t*-test).

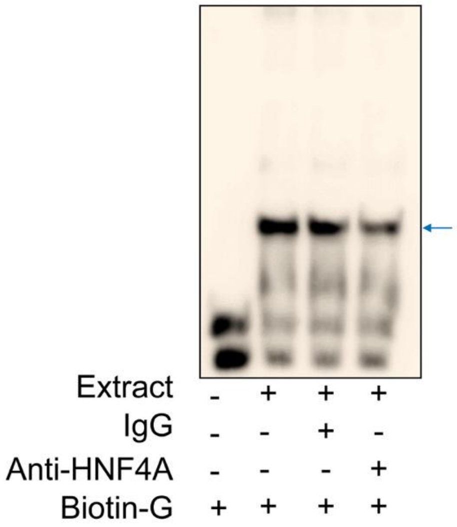


**Supplementary Figure 11 The blocking EMSA of transcription factor HNF4A in the HepG2 cell line.** Whether HNF4A binds to biotin-G oligo is proved in addition of anti-HNF4A antibody into the nuclear extracts of the HepG2 cell line. IgG acts as the negative control. The light blue arrow indicates the HNF4A-biotin-G-oligo complexes. This experiment is repeated two times at least and similar results are observed.

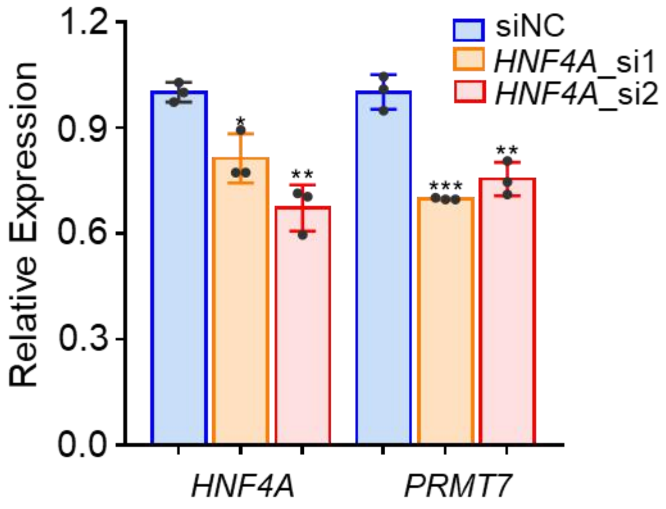


**Supplementary Figure 12 The expression of *PRMT7* is reduced after knockdown of transcription factor HNF4A in the HepG2 cell line.** qPCR detection of the relative expression of *HNF4A* and *PRMT7* after downregulation of *HNF4A* individually by two siRNAs in the HepG2 cell line. For detection of *HNF4A* expression level, $P$ = 0.0125 and 0.0013 in HNF4A_si1 or HNF4A_si2 compared to siNC. For detection of *PRMT7* expression level, $P$ = 0.0004 and $P$ = 0.0033 in HNF4A_si1 or HNF4A_si2 compared to siNC. Values are expressed as the mean ± SD, $n$ = 3. *, **, and *** mean $P$ value less than 0.05, 0.01 and 0.001, respectively (two-sided student' $t$-test).

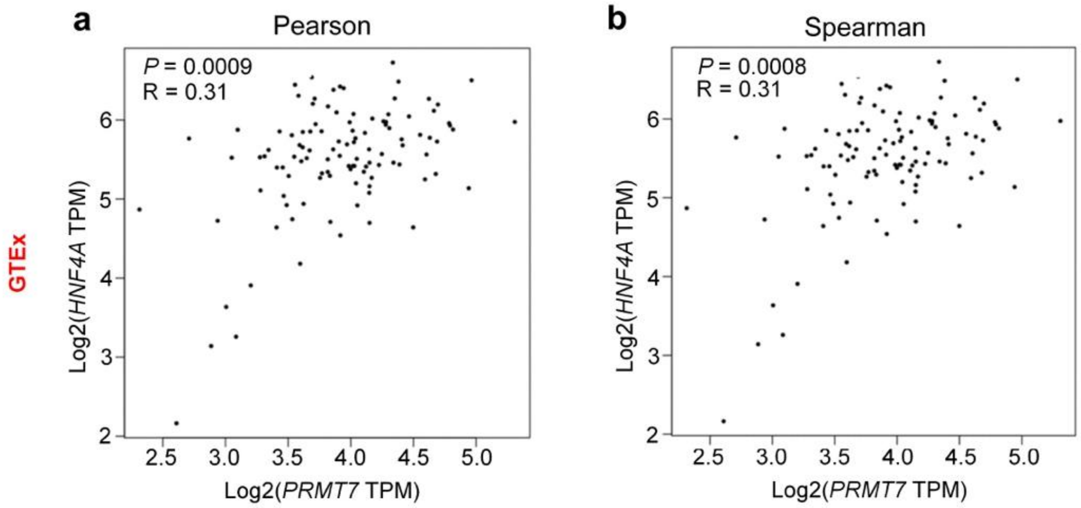


**Supplementary Figure 13 The expression of *PRMT7* and *HNF4A* have a positive correlation of in liver tissues of GTEx.** Both Pearson (**a**) and Spearman (**b**) analyses show that the *PRMT7* RNA level is positively correlated with the expression of *HNF4A* in liver tissues of GTEx provided by GEPIA website. TPM, transcripts per million.

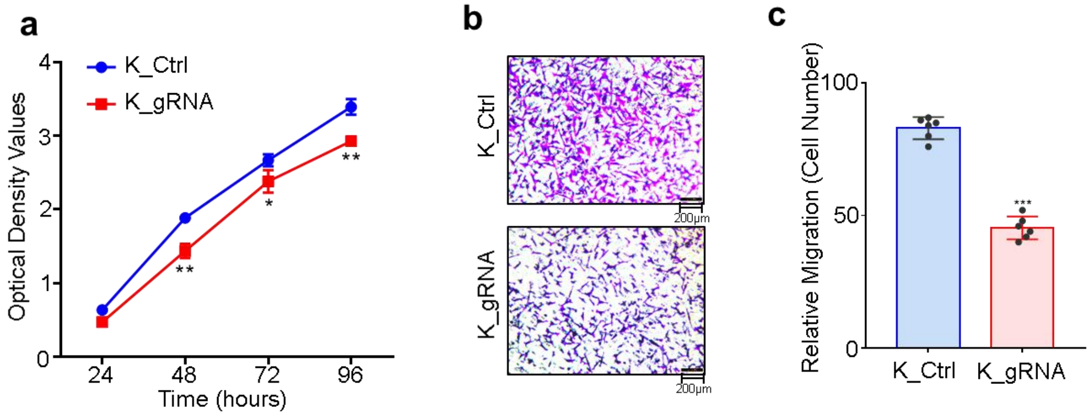


**Supplementary Figure 14 *PRMT7*-downregulated cells by CRISPRi show reduced cell growth rate and migration ability. a** CCK-8 assay for *PRMT7*-depleted cells (K_gRNA) and the control (K_Ctrl). $P$ = 0.0029 in K_gRNA compared to K_Ctrl at 96 h. **b** Transwell assay for examination of cell migration ability in cells with *PRMT7* downregulation and the control. **c** The migrated cell number counted for cells in (**b**). $P$ < 0.0001 in K_gRNA compared to K_Ctrl. Values are expressed as the mean ± SD, $n$ = 3 in **a** and $n$ = 6 in **c**. ** and *** mean $P$ value less than 0.01 and 0.001, respectively (two-sided Student' $t$-test).

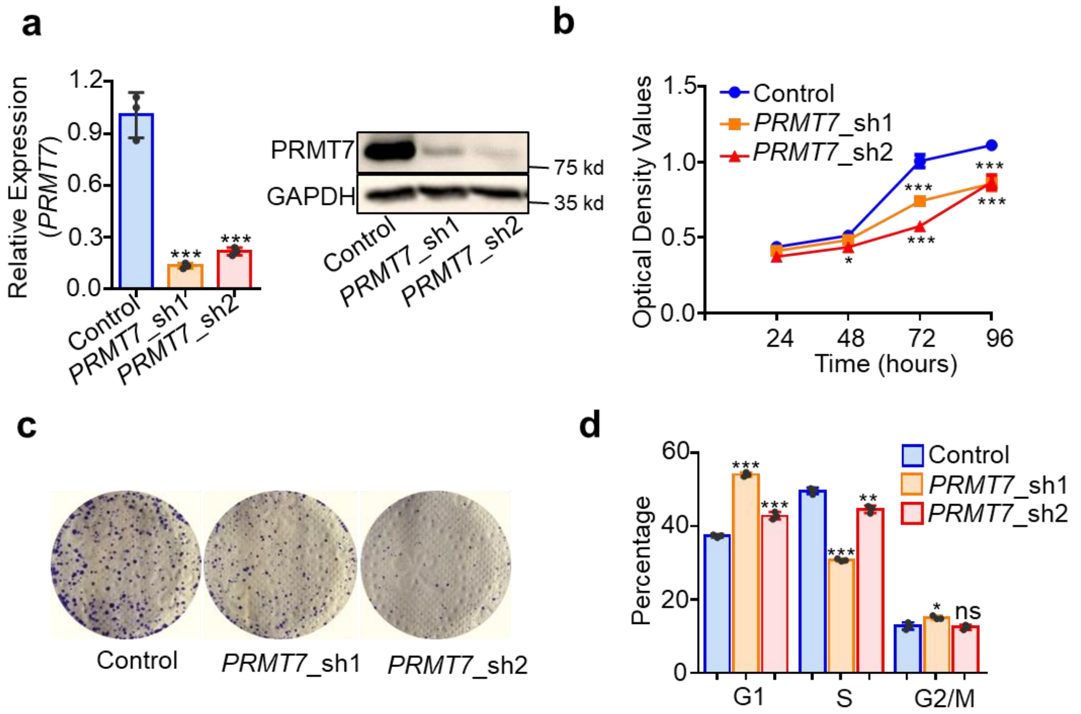


**Supplementary Figure 15 Downregulation of *PRMT7* in the HepG2 cell line reduces cell malignant phenotypes. a** RNA and protein abundance of PRMT7 detected by qPCR and western blot after knockdown of *PRMT7* in the HepG2 cell line by two shRNAs, respectively. $P$ = 0.0003 and $P$ = 0.0005 in *PRMT7*_sh1 or *PRMT7*_sh2 compared to Control. **b** CCK-8 assay for *PRMT7*-depleted cells and the control. $P < 0.0001$ in *PRMT7*_sh1 or *PRMT7*_sh2 compared to Control at 96 h. **c** Cell colony-formation assay for cells with knockdown of *PRMT7* and the control. **d** The examination of cell cycle upon the control and *PRMT7*-downregulated cells. G1 phase: $P < 0.0001$, $P = 0.0009$; S phase: $P < 0.0001$, $P = 0.0029$; G2 phase $P = 0.0023$ and $P$ = 0.6627, in *PRMT7*_sh1 or *PRMT7*_sh2 compared to Control. Values are expressed as the mean ± SD, $n = 3$ in **a**, **b**, and **d**. 'ns' means not significant; *, ** and *** mean $P$ value less than 0.05, 0.01 and 0.001, respectively (two-sided Student' *t*-test).

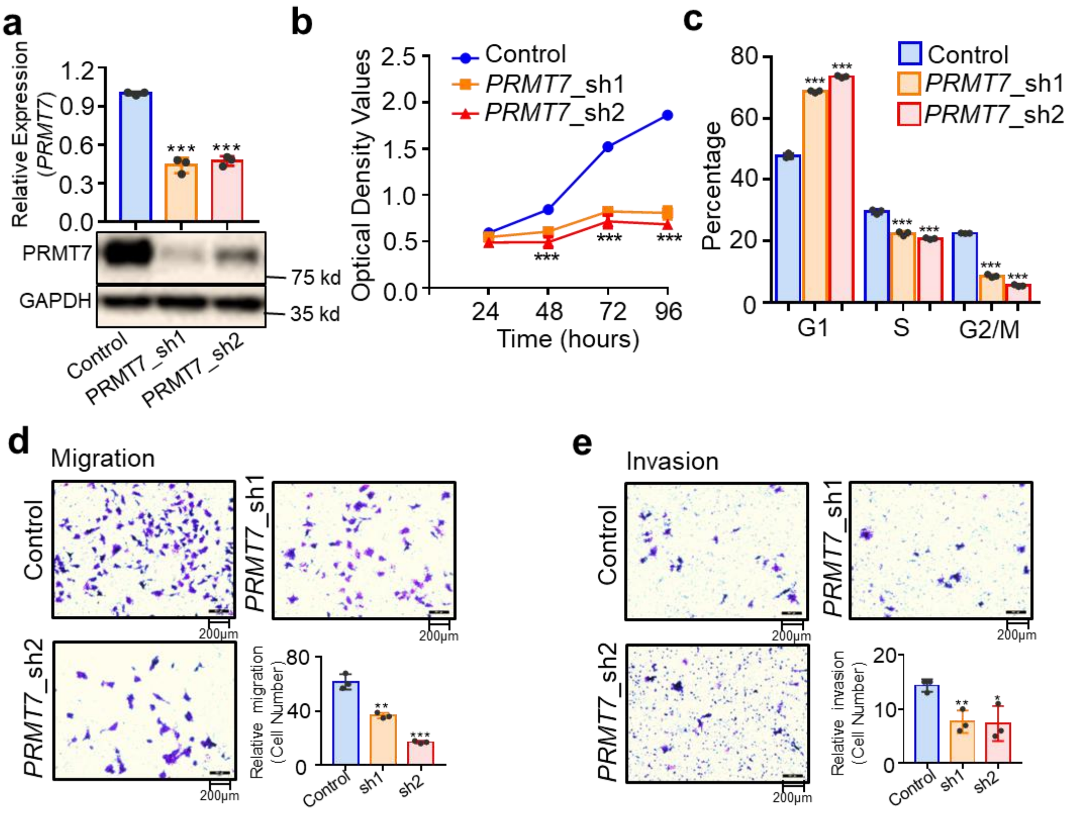


**Supplementary Figure 16 Downregulation of *PRMT7* in SMMC-7721 cell line reduces cell malignant phenotypes. a** RNA and protein abundance of PRMT7 detected by qPCR and western blot after knockdown of *PRMT7* in SMMC-7721 cell line by two shRNAs, respectively. *P* < 0.0001 in *PRMT7*_sh1 or *PRMT7*_sh2 compared to Control. **b** CCK-8 assay for *PRMT7*-depleted cells and the control. *P* < 0.0001 in *PRMT7*_sh1 or *PRMT7*_sh2 compared to Control at 96 h. **c** The examination of cell cycle upon the control and *PRMT7*-downregulated cells. *P* < 0.0001 in *PRMT7*_sh1 or *PRMT7*_sh2 compared to Control in G1, S, and G2/M phase. **d**, **e** The detection of cell migration (**d,** *P* = 0.002 and *P* = 0.0002 in *PRMT7*_sh1 or *PRMT7*_sh2 compared to Control) and invasion (**e,** in *P* = 0.0083 and *P* = 0.0238 in *PRMT7*_sh1 or *PRMT7*_sh2 compared to Control) ability for cells with knockdown of *PRMT7* and the control. Values are expressed as the mean ± SD, *n* = 3 in **a**-**e**. *, ** and *** mean *P* value less than 0.05, 0.01 and 0.001, respectively (two-sided Student' *t*-test).

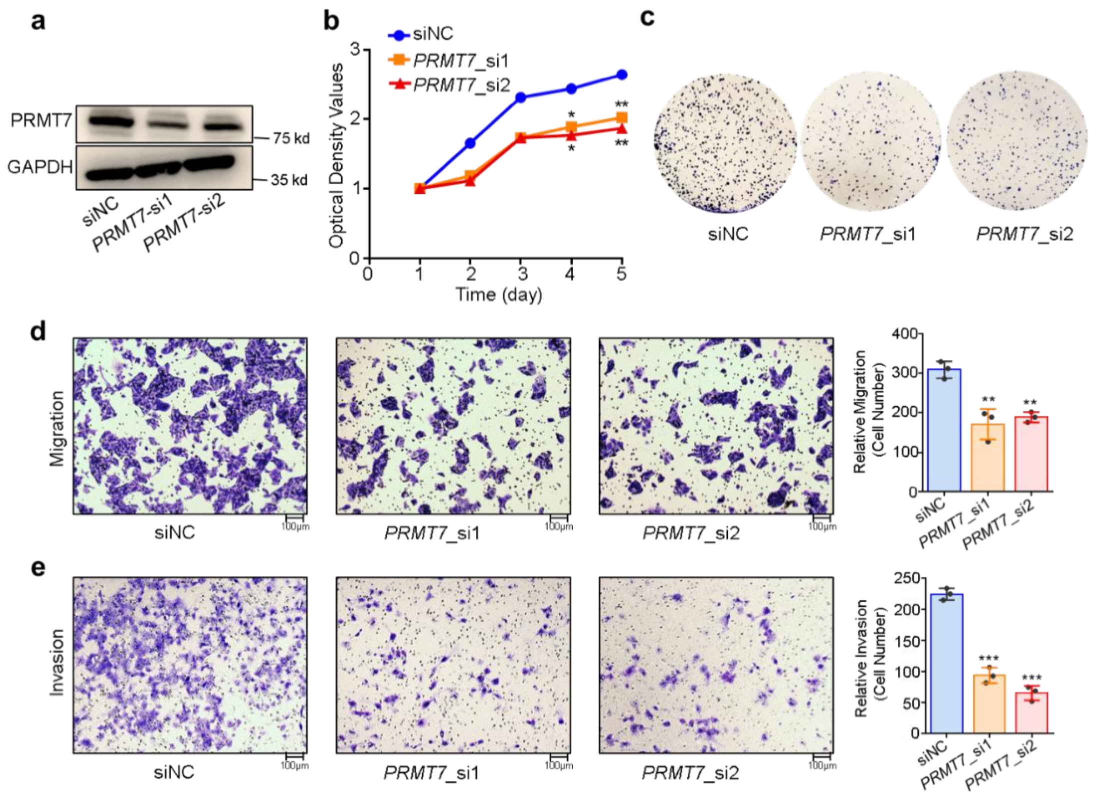


**Supplementary Figure 17 Downregulation of *PRMT7* in HepAD38 cell line reduces cell malignant phenotypes**. **a** Protein abundance of PRMT7 detected by western blot after knockdown of *PRMT7* in HepAD38 cell line by two siRNAs, respectively. **b** CCK-8 assay for *PRMT7*-depleted cells and the control. $P$ = 0.0050 and $P$ = 0.0020 in *PRMT7*_si1 or *PRMT7*_si2 compared to siNC at 120 h, respectively. **c** Cell colony formation assay for cells with knockdown of *PRMT7* and the control. **d**, **e** The detection of cell migration (**d,** $P$ = 0.0058 and $P$ = 0.0012 in *PRMT7*_si1 or *PRMT7*_si2 compared to siNC) and invasion (**e,** $P$ = 0.0001 and $P$ < 0.0001 in *PRMT7*_si1 or *PRMT7*_si2 compared to siNC) ability for cells with knockdown of *PRMT7* and the control. Values are expressed as the mean ± SD, $n$ = 3 in **b**, and **d-e**. *, ** and *** mean $P$ value less than 0.05, 0.01 and 0.001, respectively (two-sided Student' $t$-test).

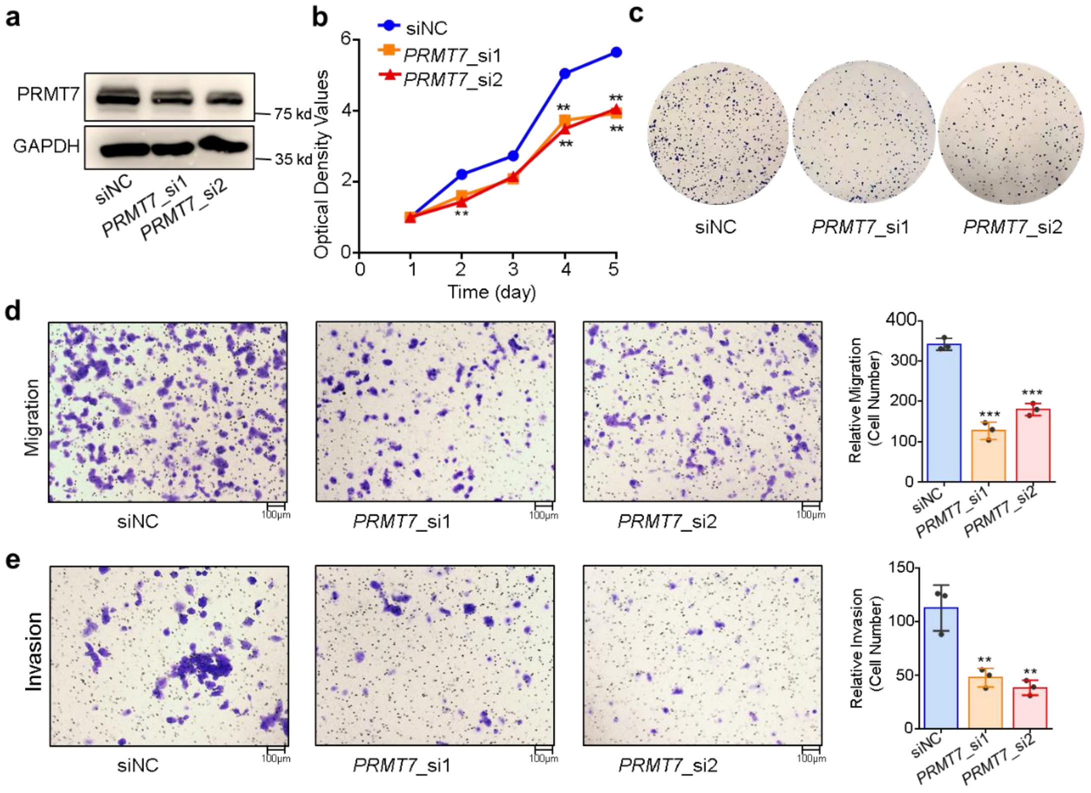


**Supplementary Figure 18 Downregulation of *PRMT7* in SNU398 cell line reduces cell malignant phenotypes**. **a** Protein abundance of PRMT7 detected by western blot after knockdown of *PRMT7* in SNU398 cell line by two siRNAs, respectively. **b** CCK-8 assay for *PRMT7*-depleted cells and the control. *P* = 0.0034 and *P* = 0.0073 in *PRMT7*_si1 or *PRMT7*_si2 compared to siNC at 120 h. **c** Cell colony formation assay for cells with knockdown of *PRMT7* and the control. **d**, **e** The detection of cell migration (**d,** *P* = 0.0001 and *P* = 0.0002 in *PRMT7*_si1 or *PRMT7*_si2 compared to siNC) and invasion (**e,** *P* = 0.0082 and *P* = 0.0046 in *PRMT7*_si1 or *PRMT7*_si2 compared to siNC) ability for cells with knockdown of *PRMT7* and the control. Values are expressed as the mean ± SD, *n* = 3 in **b**, and **d**-**e**. *, **, and *** mean *P* value less than 0.05, 0.01, and 0.001, respectively (two-sided Student' *t*-test).

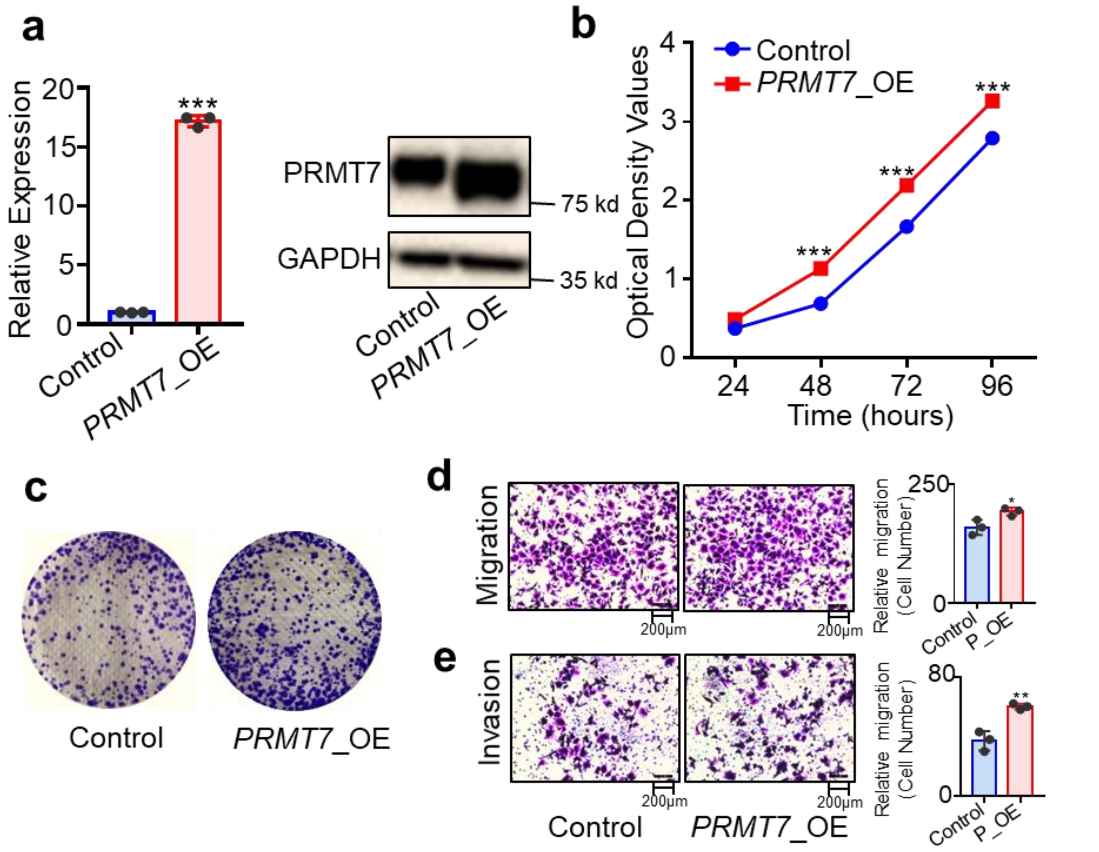


**Supplementary Figure 19 Overexpression of *PRMT7* promotes cell proliferation rate, migration, and invasion ability. a** RNA and protein abundance of PRMT7 detected by qPCR and western blot after overexpression of *PRMT7* in the QGY-7703 cell line. *P* = 0.0003 in *PRMT7*_OE compared to Control. **b** CCK-8 assay for *PRMT7*-overexpressed cells and the control. *P* < 0.0001 in *PRMT7*_OE compared to Control at 96 h. **c** Cell colony-formation assay for cells with the control and upregulation of *PRMT7*. **d**, **e** The detection of cell migration (**d,** *P* = 0.0329 in *PRMT7*_OE compared to Control.) and invasion (**e,** *P* = 0.0044 in *PRMT7*_OE compared to Control.) ability for cells with overexpression of *PRMT7* and the control. Values are expressed as the mean ± SD, *n* = 3 in **a**-**b**, and **d**-**e**. *, **, and *** mean *P* value less than 0.05, 0.01, and 0.001, respectively (two-sided Student' *t*-test).

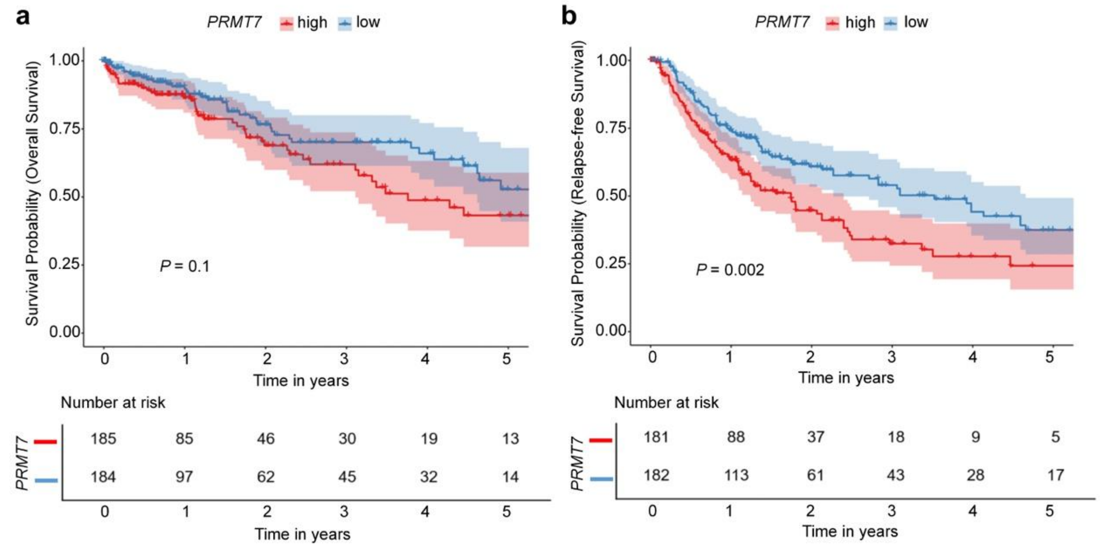


**Supplementary Figure 20 Association of *PRMT7* expression with clinical outcome of HCC patients (TCGA-LIHC). a** Association of *PRMT7* expression with overall survival of HCC patients. **b** Association of *PRMT7* expression with relapse-free survival of HCC patients. Survival curves were estimated according to the Kaplan-Meier method, and the statistical differences in the survival curves of different subgroups of subjects were analyzed using the log-rank test.

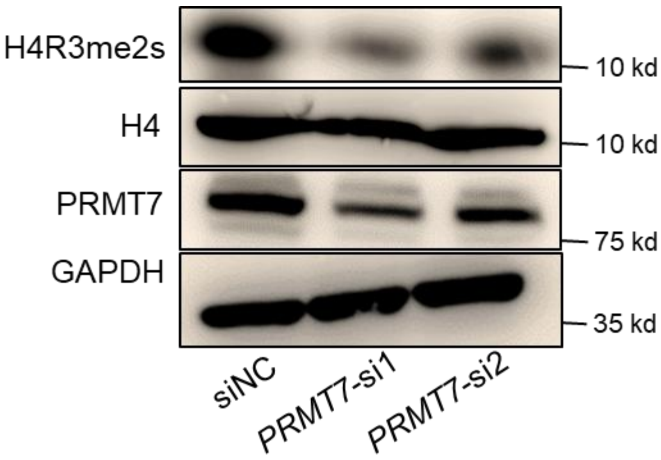


**Supplementary Figure 21 Western blot to detect H4R3me2s signal in the control and *PRMT7*-downregulated samples in HepAD38 cell line.** This experiment is replicated two times at least and similar results are observed.

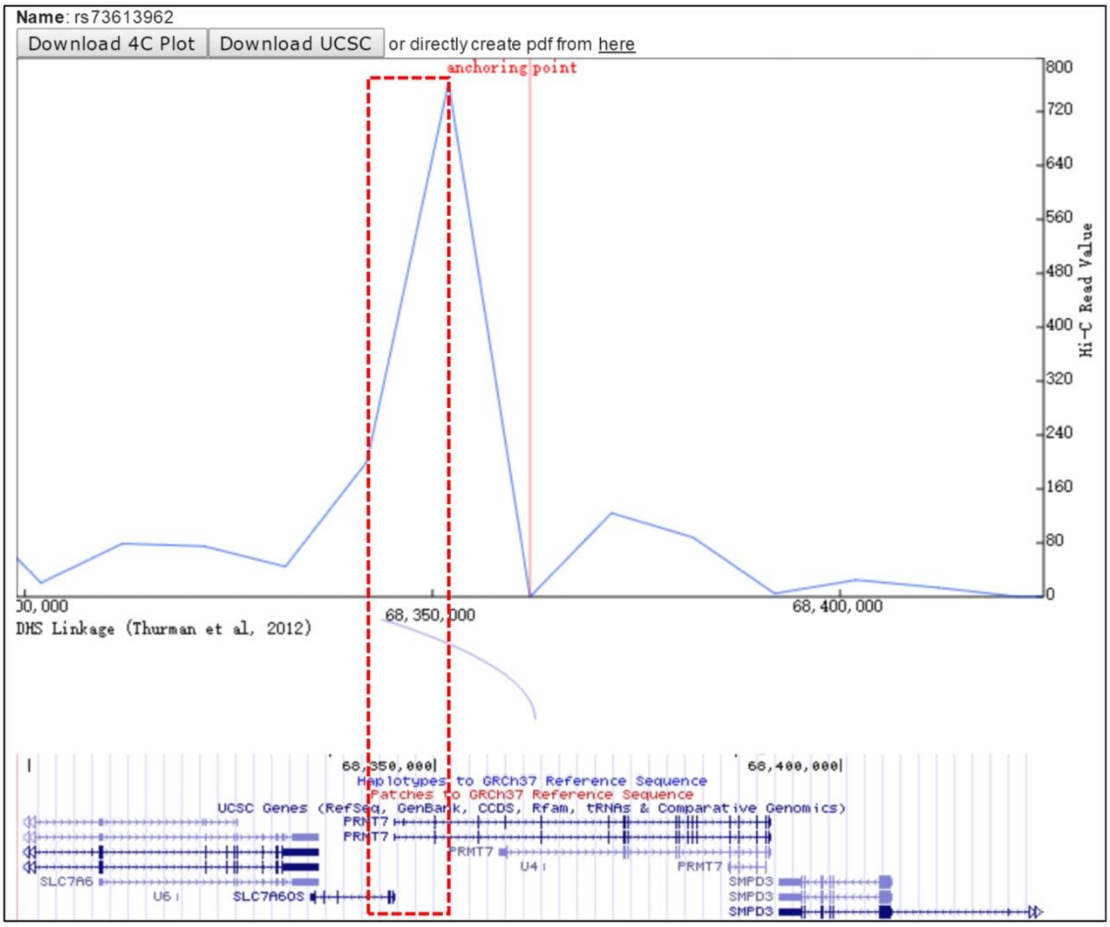


**Supplementary Figure 22 The interaction of the rs73613962-containing region with other regions upstream and downstream of this SNP site.** Visualization of the interaction between rs73613962 and other regions analyzed by Hi-C data of human liver tissue from the 3D Genome Brower[2, 3]. The anchoring point is rs73613962 in the picture. The potential interaction location of rs73613962 is highlighted by red dotted rectangle in the regions surrounding the transcription star site (TSS) of *PRMT7*.

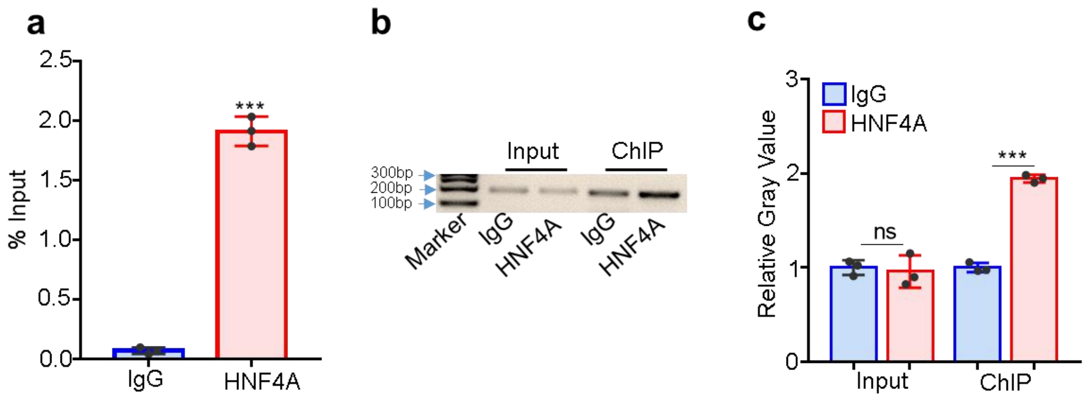


**Supplementary Figure 23 The binding of transcription factor HNF4A on the *PRMT7* promoter region in the QGY-7703 cell line. a** qPCR detection for the enrichment of the *PRMT7* promoter region upon anti-HNF4A ChIP and IgG. $P < 0.0001$ in HNF4A compared to IgG. **b** The agarose gel electrophoresis of PCR products for Input and ChIP, respectively. The light blue arrows indicate the marker bands. **c** Relative gray value of each PCR band in (**b**) analyzed by ImageJ v1.4.3.67. $P = 0.7210$ and $P < 0.0001$ in HNF4A compared to IgG in Input and ChIP, respectively. Values are expressed as the mean ± SD, $n = 3$ in **a** and **c**. 'ns' means not significant; *** means $P$ value less than 0.001 (two-sided student' $t$-test).

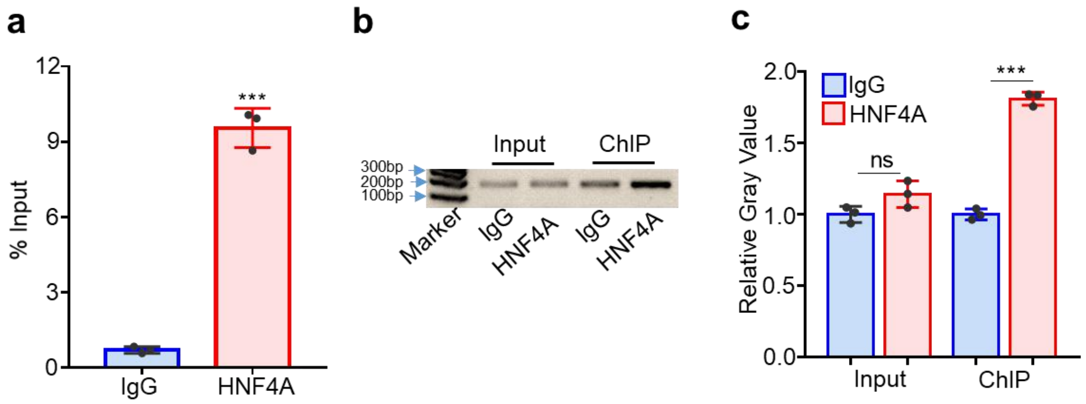


**Supplementary Figure 24 The binding of transcription factor HNF4A on the *PRMT7* promoter region in the HepG2 cell line. a** qPCR detection for the enrichment of the *PRMT7* promoter region upon anti-HNF4A ChIP and IgG. $P < 0.0001$ in HNF4A compared to IgG. **b** The agarose gel electrophoresis of PCR products for Input and ChIP, respectively. The light blue arrows indicate the marker bands. **c** Relative gray value of each PCR band in (**b**) analyzed by ImageJ v1.4.3.67. $P = 0.0854$ and $P < 0.0001$ in HNF4A compared to IgG in Input and ChIP, respectively. Values are expressed as the mean ± SD, $n = 3$ in **a** and **c**. 'ns' means not significant; *** means $P$ value less than 0.001 (two-sided student' *t*-test).

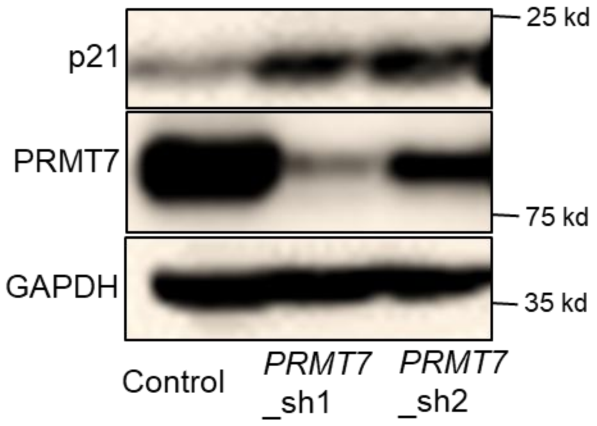


**Supplementary Figure 25 Western blot to detect p21 protein level in the control and *PRMT7*-downregulated samples in QGY-7703 cell line.** This experiment is repeated two times at least and similar results are observed.

**Supplementary Table 1 Demographic characteristics of subjects analyzed in the discovery and replication stages**

| Subjects[a] | Number | Gender, n (%) | | Age, years | | |
|---|---|---|---|---|---|---|
| | | Female | Male | Mean (SD) | ≤50, n (%) | ＞50, n (%) |
| **Discovery stage[b]** | | | | | | |
| Cases | 1161 | 263 (22.7) | 898 (77.3) | 51.6 (10.4) | 525 (45.2) | 636 (54.8) |
| Controls | 1353 | 436 (32.2) | 917 (67.8) | 48.4 (11.1) | 793 (58.6) | 560 (41.4) |
| **Replication stage 1[c]** | | | | | | |
| Cases | 576 | 91 (15.8) | 485 (82.4) | 52.0 (11.2) | 275 (47.7) | 301 (52.3) |
| Controls | 721 | 289 (40.1) | 432 (59.9) | 53.0 (13.9) | 220 (30.5) | 501 (69.5) |
| **Replication stage 2a[d]** | | | | | | |
| Cases | 1942 | 353 (18.2) | 1589 (81.8) | 51.7 (11.6) | 911 (49.5) | 1031 (50.5) |
| Controls | 2812 | 812 (28.9) | 2000 (71.1) | 46.3 (14.3) | 1655 (58.9) | 1157 (41.1) |
| **Replication stage 2b[e]** | | | | | | |
| Cases | 393 | 60 (15.3) | 333 (84.7) | 54.4 (10.3) | 134 (34.1) | 259 (65.9) |
| Controls | 1314 | 317 (24.1) | 997 (75.9) | 33.6 (10.9) | 1212 (92.2) | 102 (7.8) |
| **Replication stage 2c[f]** | | | | | | |
| Cases | 826 | 178 (21.5) | 648 (78.5) | 47.8 (12.4) | 522 (63.2) | 304 (36.8) |
| Controls | 860 | 191 (22.2) | 669 (77.8) | 46.1 (12.4) | 572 (66.5) | 288 (33.5) |

SD, standard deviation.

[a]All the subjects including HCC cases and non-HCC controls of the five populations are chronic HBV carriers. [b]The subjects in the discovery stage were recruited from Qidong, Jiangsu province in East China. [c]The subjects in the replication stage 1 were recruited from Shanghai in East China. [d]The subjects in the replication stage 2a were recruited from Shanghai and Jiangsu province in East China; [e]The subjects in the replication stage 2b were recruited from Beijing and Shangdong province in North China; [f]The subjects in the replication stage 2c were recruited from Guangxi province in South China.

**Supplementary Table 2 Summary of the six SNPs selected from the discovery stage for validation in the replication stage 1**

| Chr. | Position | Gene | Region | SNP | Allele[a] | Study | MAF | | OR (95% CI)[b] | $P^b$ |
|---|---|---|---|---|---|---|---|---|---|---|
| | | | | | | | Cases | Controls | | |
| 4 | 69604491 | *UGT2B15* | Intergenic | rs72651930 | A/G | Discovery | 0.13 | 0.09 | 1.42 (1.18-1.71) | 2.73E-04 |
| | | | | | | Replication 1 | 0.12 | 0.11 | 1.15 (0.90-1.47) | 2.72E-01 |
| 4 | 69638061 | *UGT2B10* | Intergenic | rs4694072[c] | C/T | Discovery | 0.13 | 0.10 | 140 (1.17-1.69) | 2.91E-04 |
| | | | | | | Replication 1 | — | — | — | — |
| 6 | 30065298 | *TRIM31* | Intergenic | rs9261395 | A/C | Discovery | 0.27 | 0.30 | 0.81 (0.71-0.92) | 8.57E-04 |
| | | | | | | Replication 1 | 0.45 | 0.44 | 1.06 (0.91-1.24) | 4.27E-01 |
| 16 | 68361974 | *PRMT7* | Intron | rs73613962 | G/T | Discovery | 0.09 | 0.06 | 1.47 (1.18-1.83) | 6.04E-04 |
| | | | | | | Replication 1 | 0.08 | 0.06 | 1.40 (1.03-1.92) | 3.31E-02 |
| 22 | 19420306 | *MRPL40* | Intron | rs12165902 | G/C | Discovery | 0.14 | 0.11 | 1.39 (1.17-1.65) | 2.23E-04 |
| | | | | | | Replication 1 | 0.11 | 0.13 | 0.77 (0.60-0.99) | 4.11E-02 |
| 22 | 36791264 | *TXN2* | Intergenic | rs74702984 | G/C | Discovery | 0.08 | 0.11 | 0.70 (0.57-0.86) | 5.49E-04 |
| | | | | | | Replication 1 | 0.12 | 0.09 | 1.25 (0.97-1.61) | 8.13E-02 |

Chr., chromosome; SNP, single nucleotide polymorphism; MAF, minor allele frequency; OR, odds ratio; CI, confidence interval.
[a]Minor allele/major allele. [b]The data was analyzed using logistic regression with adjustment for gender and age. [c]Since this SNP is in high linkage disequilibrium with rs72651930 ($r^2 > 0.9$), it was not genotyped in replication stage 1 samples.

## Supplementary References


1. Paulsen, M.T. *et al.* Use of Bru-Seq and BruChase-Seq for genome-wide assessment of the synthesis and stability of RNA. *Methods* **67**, 45-54 (2014).
2. Leung, D. *et al.* Integrative analysis of haplotype-resolved epigenomes across human tissues. *Nature* **518**, 350-354 (2015).
3. Wang, Y. *et al.* The 3D Genome Browser: a web-based browser for visualizing 3D genome organization and long-range chromatin interactions. *Genome Biol* **19**, 151 (2018).

## Source Data

## Supplementary Figure 2

### Supplementary Figure 2b

| | Control | Enhancer-T | Enhancer-G | | Control | Enhancer-T | Enhancer-G |
|---|---|---|---|---|---|---|---|
| | 840 | 8367 | 9558 | | 3.6681 | 33.7379 | 62.4706 |
| | 824 | 9629 | 10860 | | 2.5046 | 34.7617 | 57.7660 |
| Firefly | 970 | 7715 | 8760 | Firefly/Renilla | 3.9113 | 50.4248 | 54.7500 |
| | 229 | 248 | 153 | | | | |
| | 329 | 277 | 188 | | | | |
| Renilla | 248 | 153 | 160 | | | | |

### Supplementary Figure 2c

| | Control | Enhancer-T | Enhancer-G | | Control | Enhancer-T | Enhancer-G |
|---|---|---|---|---|---|---|---|
| | 842 | 5372 | 4331 | | 1.5506 | 8.7921 | 11.1624 |
| | 752 | 4226 | 4297 | | 1.7014 | 8.7495 | 11.7726 |
| Firefly | 724 | 4741 | 3535 | Firefly/Renilla | 1.4480 | 8.0220 | 10.3971 |
| | 543 | 611 | 388 | | | | |
| | 442 | 483 | 365 | | | | |
| Renilla | 500 | 591 | 340 | | | | |

### Supplementary Figure 2e

| | Control | Enhancer-T | Enhancer-G | | Control | Enhancer-T | Enhancer-G |
|---|---|---|---|---|---|---|---|
| | 840 | 4319 | 4369 | | 3.6681 | 20.0884 | 27.1366 |
| | 824 | 3971 | 3655 | | 2.5046 | 17.2652 | 28.7795 |
| Firefly | 970 | 3962 | 3968 | Firefly/Renilla | 3.9113 | 17.8468 | 26.8108 |
| | 229 | 215 | 161 | | | | |
| | 329 | 230 | 127 | | | | |
| Renilla | 248 | 222 | 148 | | | | |

### Supplementary Figure 2f

| | Control | Enhancer-T | Enhancer-G | | Control | Enhancer-T | Enhancer-G |
|---|---|---|---|---|---|---|---|
| | 842 | 5347 | 3848 | | 1.5506 | 16.7094 | 21.1429 |
| | 752 | 4673 | 4100 | | 1.7014 | 15.5249 | 17.7489 |
| Firefly | 724 | 3813 | 4425 | Firefly/Renilla | 1.4480 | 15.0711 | 22.5765 |
| | 543 | 320 | 182 | | | | |
| | 442 | 301 | 231 | | | | |
| Renilla | 500 | 253 | 196 | | | | |

**Supplementary Figure 4**

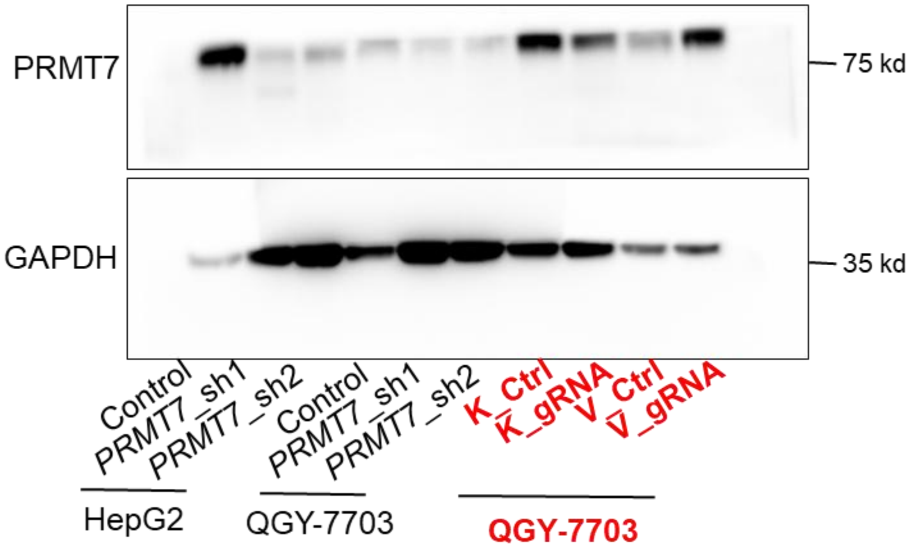


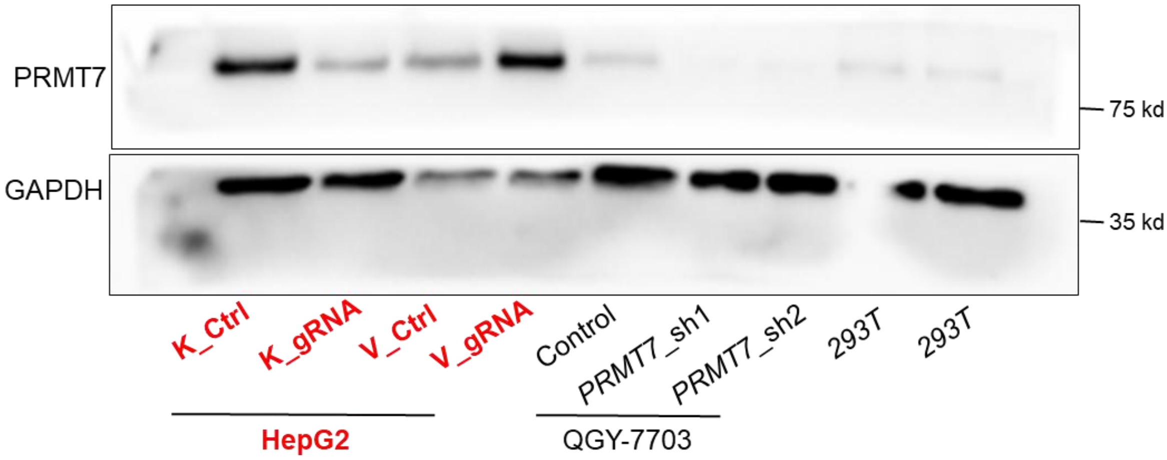

**Supplementary Figure 5**

ct values

| K_Ctrl | | K_gRNA | | V_Ctrl | | V_gRNA | |
|---|---|---|---|---|---|---|---|
| GAPDH | PRMT7 | GAPDH | PRMT7 | GAPDH | PRMT7 | GAPDH | PRMT7 |
| 20.52 | 27.33 | 19.47 | 27.43 | 19.91 | 26.69 | 19.58 | 26.44 |
| 20.37 | 27.4 | 19.51 | 27.6 | 19.55 | 26.7 | 19.66 | 26.42 |
| 20.38 | 27.26 | 19.48 | 27.42 | 19.82 | 26.87 | 20.03 | 26.43 |

Relative expression of *PRMT7* normalized to GAPDH

| K_Ctrl | K_gRNA | V_Ctrl | V_gRNA |
|---|---|---|---|
| 1.0000 | 0.4874 | 1.0449 | 1.2287 |
| 0.9526 | 0.4332 | 1.0377 | 1.2458 |
| 1.0497 | 0.4908 | 0.9223 | 1.2372 |

**Supplementary Figure 8**

Supplementary Figure 8a

ct values

| IgG_Input | HNF4A_Input | IgG | HNF4A |
|---|---|---|---|
| 18.89 | 18.85 | 23.65 | 19.35 |
| 18.90 | 18.89 | 23.59 | 19.31 |
| 18.83 | 18.91 | 23.64 | 19.48 |

% Input

| % Input_IgG | % Input_HNF4A |
|---|---|
| 0.3691 | 7.0711 |
| 0.3874 | 7.4742 |
| 0.3565 | 6.7362 |

Supplementary Figure 8b

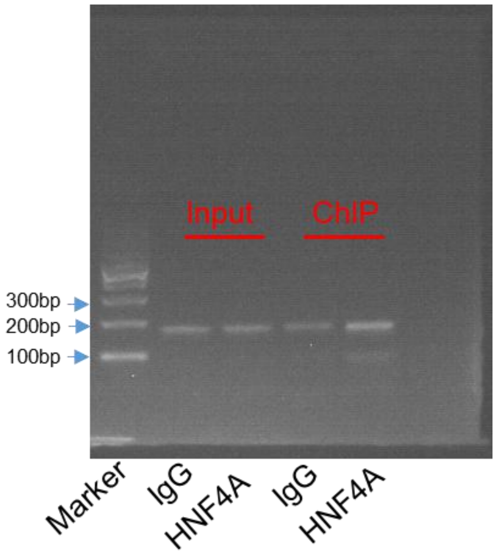


Supplementary Figure 8c

Grey values

| IgG_Input | HNF4A_Input | IgG | HNF4A |
|---|---|---|---|
| 3257 | 3141 | 2400 | 6129 |
| 3119 | 3459 | 2431 | 6045 |
| 3219 | 3449 | 2290 | 6034 |

Relative grey values

| IgG_Input | HNF4A_Input | IgG | HNF4A |
|---|---|---|---|
| 0.9820 | 1.0285 | 0.9890 | 2.5289 |
| 1.0254 | 1.0740 | 0.9764 | 2.4966 |
| 0.9936 | 1.0406 | 1.0365 | 2.6504 |

**Supplementary Figure 9**

ct values

| IgG_Input | HNF4A_Input | IgG | HNF4A |
|---|---|---|---|
| 32.23 | 31.55 | 32.75 | 32.45 |
| 32.40 | 32.27 | 33.99 | 32.63 |
| 31.82 | 32.65 | 33.07 | 32.90 |

% Input

| % Input_IgG | % Input_HNF4A |
|---|---|
| 6.9737 | 5.3589 |
| 3.3217 | 7.7916 |
| 4.2045 | 8.4090 |

**Supplementary Figure 10**

ct values

| | IgG_Input | HNF4A_Input | IgG | HNF4A |
|---|---|---|---|---|
| | 20.10 | 20.13 | 26.52 | 26.70 |
| | 20.14 | 20.11 | 26.69 | 26.45 |
| Control | 20.17 | 20.17 | 26.52 | 26.85 |
| | IgG_Input | HNF4A_Input | IgG | HNF4A |
| | 9.80 | 9.89 | 17.79 | 15.52 |
| | 9.83 | 9.80 | 17.73 | 15.53 |
| TT | 9.78 | 9.91 | 17.83 | 15.53 |
| | IgG_Input | HNF4A_Input | IgG | HNF4A |
| | 9.61 | 9.55 | 18.43 | 15.25 |
| | 9.60 | 9.67 | 18.17 | 15.34 |
| GG | 9.61 | 9.54 | 18.17 | 15.26 |

Relative enrichment

| Control | TT | GG |
|---|---|---|
| 0.9013 | 5.1337 | 8.6939 |
| 1.1567 | 4.5002 | 7.4643 |
| 0.7955 | 5.3889 | 7.1602 |

**Supplementary Figure 12**

ct values

| siNC | | | HNF4A_si1 | | | HNF4A_si2 | | |
|---|---|---|---|---|---|---|---|---|
| GAPDH | HNF4A | PRMT7 | GAPDH | HNF4A | PRMT7 | GAPDH | HNF4A | PRMT7 |
| 19.04 | 30.71 | 25.35 | 18.89 | 31.02 | 25.86 | 18.82 | 31.13 | 25.76 |
| 18.77 | 30.63 | 25.3 | 19.05 | 31.23 | 25.85 | 18.69 | 31.39 | 25.65 |
| 18.64 | 30.67 | 25.44 | 19.08 | 31.23 | 25.86 | 18.87 | 31.15 | 25.83 |

Relative expression of *HNF4A* and *PRMT7* normalized to GAPDH

| siNC | | HNF4A_si1 | | HNF4A_si2 | |
|---|---|---|---|---|---|
| HNF4A | PRMT7 | HNF4A | PRMT7 | HNF4A | PRMT7 |
| 0.9727 | 1.0093 | 0.8935 | 0.6964 | 0.7143 | 0.7464 |
| 1.0281 | 1.0449 | 0.7725 | 0.7012 | 0.5965 | 0.8055 |
| 1.0000 | 0.9482 | 0.7725 | 0.6964 | 0.7045 | 0.7110 |

**Supplementary Figure 14**

Supplementary Figure 14a

| | 24 h | | | 48 h | | | 72 h | | | 96 h | | |
|---|---|---|---|---|---|---|---|---|---|---|---|---|
| K_Ctrl | 0.599 | 0.669 | 0.630 | 1.829 | 1.901 | 1.931 | 2.611 | 2.765 | 2.642 | 3.503 | 3.401 | 3.290 |
| K_gRNA | 0.448 | 0.507 | 0.456 | 1.332 | 1.463 | 1.518 | 2.480 | 2.462 | 2.209 | 3.003 | 2.910 | 2.882 |

Supplementary Figure 14c

Relative migration (cell number)

| | | | | | | |
|---|---|---|---|---|---|---|
| K_Ctrl | 86 | 80 | 87 | 76 | 85 | 84 |
| K_gRNA | 48 | 40 | 42 | 44 | 46 | 52 |

**Supplementary Figure 15**

Supplementary Figure 15a

qPCR

ct values

| Control | | PRMT7_sh1 | | PRMT7_sh2 | |
|---|---|---|---|---|---|
| GAPDH | PRMT7 | GAPDH | PRMT7 | GAPDH | PRMT7 |
| 16.49 | 25.47 | 16.65 | 28.2 | 16.33 | 27.50 |
| 16.46 | 25.18 | 16.75 | 28.53 | 16.32 | 27.20 |
| 16.29 | 25.10 | 16.44 | 28.36 | 16.24 | 27.35 |

Relative expression of PRMT7 normalized to GAPDH

| Control | PRMT7_sh1 | PRMT7_sh2 |
|---|---|---|
| 0.8586 | 0.1480 | 0.1938 |
| 1.0497 | 0.1178 | 0.2386 |
| 1.1096 | 0.1325 | 0.2151 |

Western blot

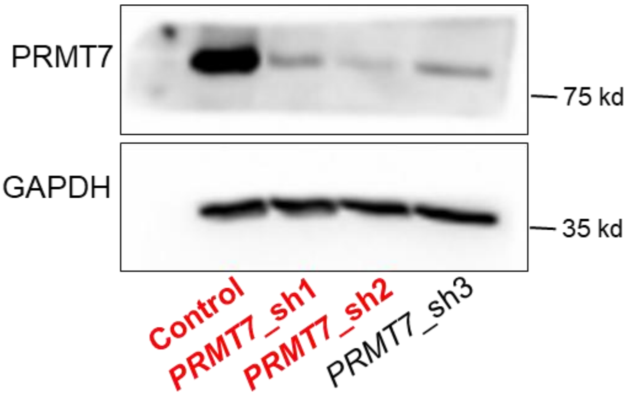


Supplementary Figure 15b

| | 24 h | | | 48 h | | | 72 h | | | 96 h | | |
|---|---|---|---|---|---|---|---|---|---|---|---|---|
| Control | 0.444 | 0.446 | 0.422 | 0.494 | 0.513 | 0.533 | 0.997 | 0.972 | 1.053 | 1.130 | 1.090 | 1.119 |
| PRMT7_sh1 | 0.403 | 0.431 | 0.401 | 0.491 | 0.482 | 0.480 | 0.724 | 0.742 | 0.757 | 0.816 | 0.849 | 0.908 |
| PRMT7_sh2 | 0.367 | 0.378 | 0.377 | 0.42 | 0.452 | 0.436 | 0.611 | 0.544 | 0.571 | 0.906 | 0.882 | 0.813 |

Supplementary Figure 15d

| Control (%) | | | PRMT7_sh1 (%) | | | PRMT7_sh2 (%) | | |
|---|---|---|---|---|---|---|---|---|
| G1 | S | G2/M | G1 | S | G2/M | G1 | S | G2/M |
| 37.53 | 48.69 | 13.78 | 54.05 | 31.20 | 14.75 | 43.93 | 43.53 | 12.53 |
| 36.92 | 50.31 | 12.76 | 54.73 | 30.44 | 14.83 | 42.76 | 45.51 | 11.73 |
| 37.70 | 50.00 | 11.82 | 53.58 | 30.73 | 15.7 | 41.93 | 44.95 | 13.12 |

**Supplementary Figure 16**

Supplementary Figure 16a

qPCR

ct values

| Control | | PRMT7_sh1 | | PRMT7_sh2 | |
|---|---|---|---|---|---|
| GAPDH | PRMT7 | GAPDH | PRMT7 | GAPDH | PRMT7 |
| 16.15 | 25.60 | 16.20 | 27.48 | 16.41 | 27.03 |
| 16.31 | 25.54 | 16.27 | 27.32 | 16.17 | 26.78 |
| 16.30 | 25.71 | 16.12 | 27.40 | 16.32 | 26.68 |

Relative expression of PRMT7 normalized to GAPDH

| Control | PRMT7_sh1 | PRMT7_sh2 |
|---|---|---|
| 1.0116 | 0.2640 | 0.3869 |
| 1.0546 | 0.2950 | 0.4601 |
| 0.9374 | 0.2791 | 0.4931 |

Western blot

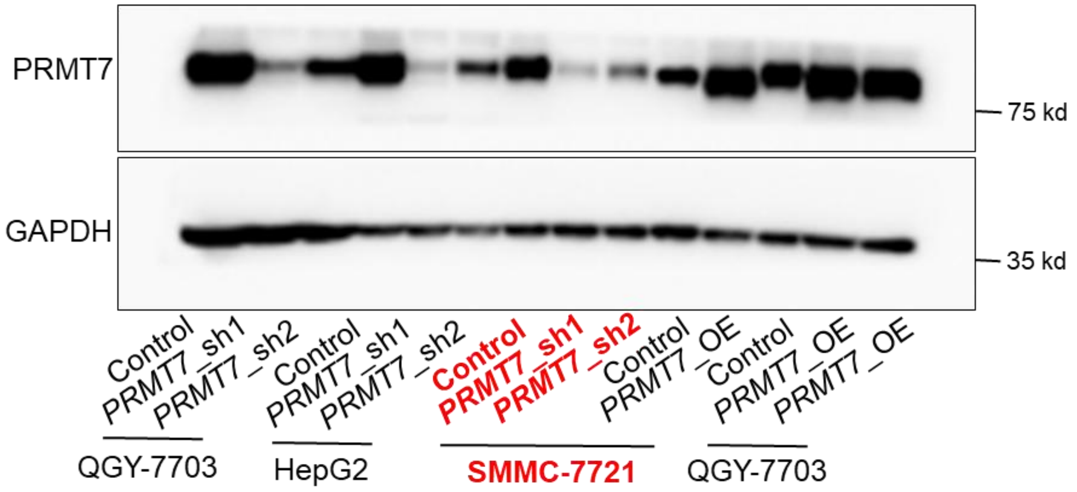


Supplementary Figure 16b

| | 24 h | | | 48 h | | | 72 h | | | 96 h | | |
|---|---|---|---|---|---|---|---|---|---|---|---|---|
| Control | 0.589 | 0.586 | 0.609 | 0.836 | 0.867 | 0.837 | 1.472 | 1.555 | 1.535 | 1.909 | 1.862 | 1.813 |
| PRMT7_sh1 | 0.567 | 0.545 | 0.536 | 0.603 | 0.627 | 0.593 | 0.772 | 0.833 | 0.870 | 0.822 | 0.740 | 0.866 |
| PRMT7_sh2 | 0.496 | 0.479 | 0.502 | 0.528 | 0.527 | 0.427 | 0.796 | 0.661 | 0.695 | 0.692 | 0.658 | 0.707 |

Supplementary Fig. 16c

| Control (%) | | | PRMT7_sh1 (%) | | | PRMT7_sh2 (%) | | |
|---|---|---|---|---|---|---|---|---|
| G1 | S | G2/M | G1 | S | G2/M | G1 | S | G2/M |

| | | | | | | | | |
|---|---|---|---|---|---|---|---|---|
| 47.14 | 68.99 | 73.13 | 30.16 | 21.76 | 20.92 | 22.69 | 9.24 | 5.95 |
| 48.70 | 68.27 | 73.57 | 28.79 | 23.05 | 21.16 | 22.52 | 8.68 | 5.28 |

Supplementary Figure 16d

Relative migration (cell number)

| Control | PRMT7_sh1 | PRMT7_sh12 |
|---|---|---|
| 60 | 39 | 16 |
| 57 | 36 | 17 |
| 68 | 35 | 18 |

Supplementary Figure 16e

Relative invasion (cell number)

| Control | PRMT7_sh1 | PRMT7_sh12 |
|---|---|---|
| 13 | 10 | 11 |
| 15 | 6 | 5 |
| 15 | 7 | 6 |

**Supplementary Figure 17**

Supplementary Figure 17a

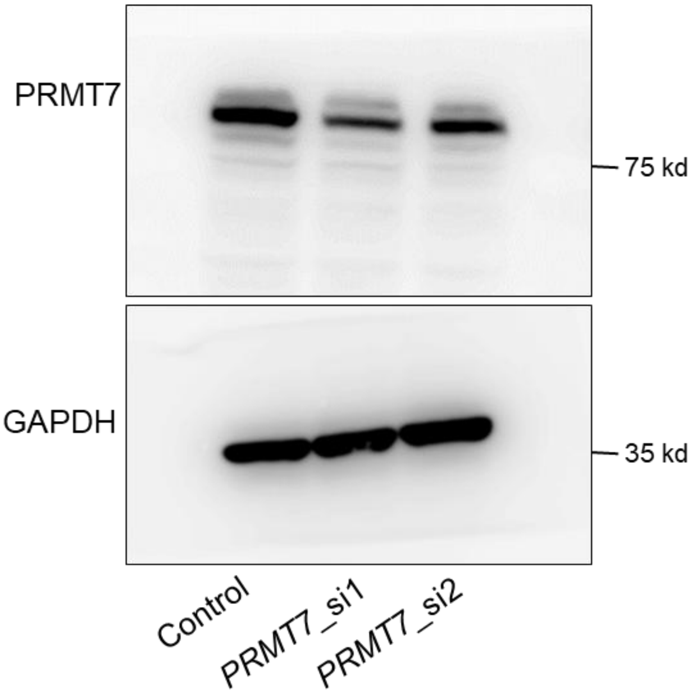


Supplementary Figure 17b

| | siNC | | | PRMT7_si1 | | | PRMT7_si2 | | |
|---|---|---|---|---|---|---|---|---|---|
| 1 day | 1.0000 | 1.0000 | 1.0000 | 1.0000 | 1.0000 | 1.0000 | 1.0000 | 1.0000 | 1.0000 |
| 2 day | 1.7590 | 1.7587 | 1.4362 | 1.1941 | 1.3270 | 1.0402 | 1.2361 | 1.0107 | 1.0955 |
| 3 day | 2.3440 | 2.6654 | 1.9274 | 1.7819 | 1.6894 | 1.7078 | 1.7672 | 1.7106 | 1.7049 |
| 4 day | 2.5812 | 2.6222 | 2.1037 | 1.8238 | 1.9550 | 1.8805 | 1.7925 | 1.8939 | 1.6028 |
| 5 day | 2.7521 | 2.7440 | 2.4277 | 1.9603 | 2.0685 | 2.0272 | 1.8553 | 1.8467 | 1.8954 |

Supplementary Figure 17d

Relative migration (cell number)

| Control | PRMT7_si1 | PRMT7_si2 |
|---|---|---|
| 329 | 189 | 176 |
| 312 | 127 | 189 |
| 286 | 198 | 202 |

Supplementary Figure 17e

Relative invasion (cell number)

| Control | PRMT7_si1 | PRMT7_si2 |
|---|---|---|
| 225 | 93 | 69 |
| 215 | 107 | 75 |
| 234 | 82 | 52 |

**Supplementary Figure 18**

Supplementary Figure 18a

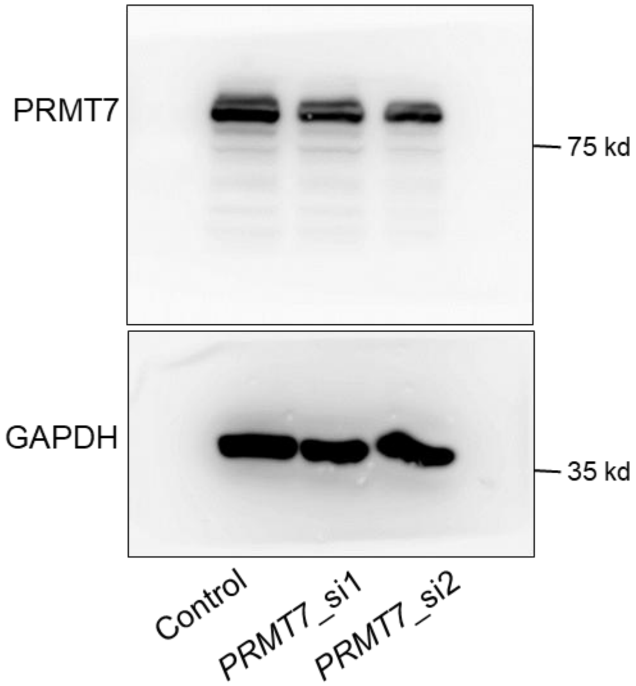


Supplementary Figure 18b

| | siNC | | | PRMT7_si1 | | | PRMT7_si2 | | |
|---|---|---|---|---|---|---|---|---|---|
| 1 day | 1.0000 | 1.0000 | 1.0000 | 1.0000 | 1.0000 | 1.0000 | 1.0000 | 1.0000 | 1.0000 |
| 2 day | 2.4026 | 2.1185 | 2.1053 | 1.4774 | 2.0407 | 1.3080 | 1.5894 | 1.2749 | 1.4516 |
| 3 day | 2.3333 | 2.6640 | 3.1989 | 2.0154 | 1.8839 | 2.3477 | 2.0196 | 2.2118 | 2.2187 |
| 4 day | 5.3076 | 5.2192 | 4.6122 | 3.8049 | 3.6527 | 3.7455 | 3.5860 | 3.3592 | 3.5310 |
| 5 day | 6.0934 | 5.6981 | 5.1454 | 3.8864 | 3.9605 | 3.9568 | 4.3620 | 3.9601 | 3.8509 |

Supplementary Figure 18d

Relative migration (cell number)

| Control | PRMT7_si1 | PRMT7_si2 |
|---|---|---|
| 331 | 130 | 195 |
| 359 | 104 | 180 |
| 336 | 147 | 165 |

Supplementary Figure 18e

Relative invasion (cell number)

| Control | PRMT7_si1 | PRMT7_si2 |
|---|---|---|
| 88 | 55 | 39 |
| 124 | 50 | 31 |
| 126 | 38 | 45 |

**Supplementary Figure 19**

Supplementary Figure 19a

qPCR

ct values

| Control | | PRMT7_OE | |
|---|---|---|---|
| GAPDH | PRMT7 | GAPDH | PRMT7 |
| 18.76 | 27.56 | 19.33 | 23.98 |
| 18.88 | 27.64 | 19.3 | 23.98 |
| 18.74 | 27.56 | 19.31 | 24.05 |

Relative expression of PRMT7 normalized to GAPDH

| Control | PRMT7_OE |
|---|---|
| 1.0187 | 17.4676 |
| 0.9637 | 17.4676 |
| 1.0187 | 16.6403 |

Western blot

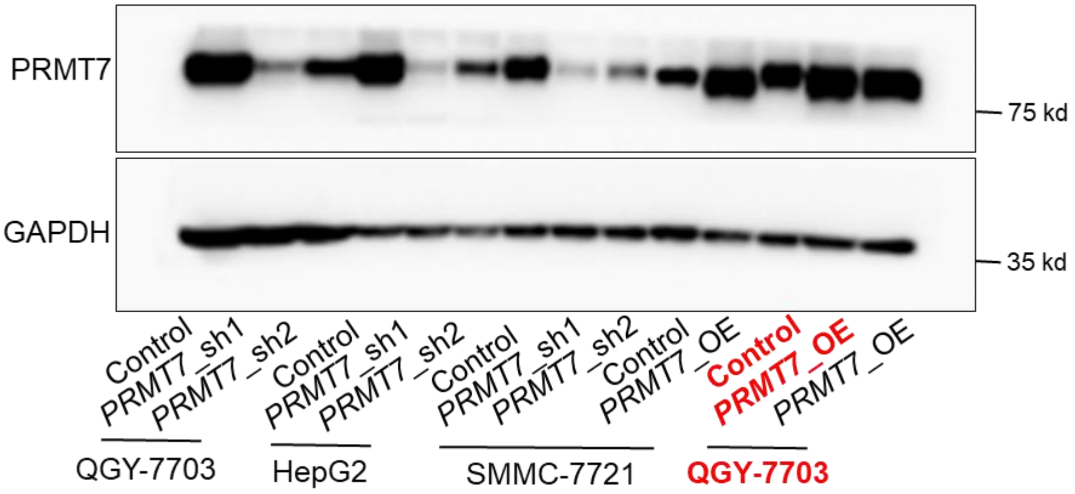


Supplementary Figure 19b

| | 24 h | | | 48 h | | | 72 h | | | 96 h | | |
|---|---|---|---|---|---|---|---|---|---|---|---|---|
| Control | 0.380 | 0.368 | 0.378 | 0.685 | 0.638 | 0.753 | 1.619 | 1.680 | 1.704 | 2.804 | 2.803 | 2.772 |
| PRMT7_OE | 0.503 | 0.503 | 0.474 | 1.119 | 1.188 | 1.096 | 2.188 | 2.149 | 2.240 | 3.323 | 3.277 | 3.188 |

Supplementary Figure 19d

Relative migration (cell number)

| Control | PRMT7_OE |
|---|---|
| 176 | 196 |
| 144 | 200 |
| 160 | 184 |

Supplementary Figure 19e

Relative invasion (cell number)

| Control | PRMT7_OE |
|---|---|
| 38 | 62 |
| 43 | 58 |
| 30 | 60 |

**Supplementary Figure 21**

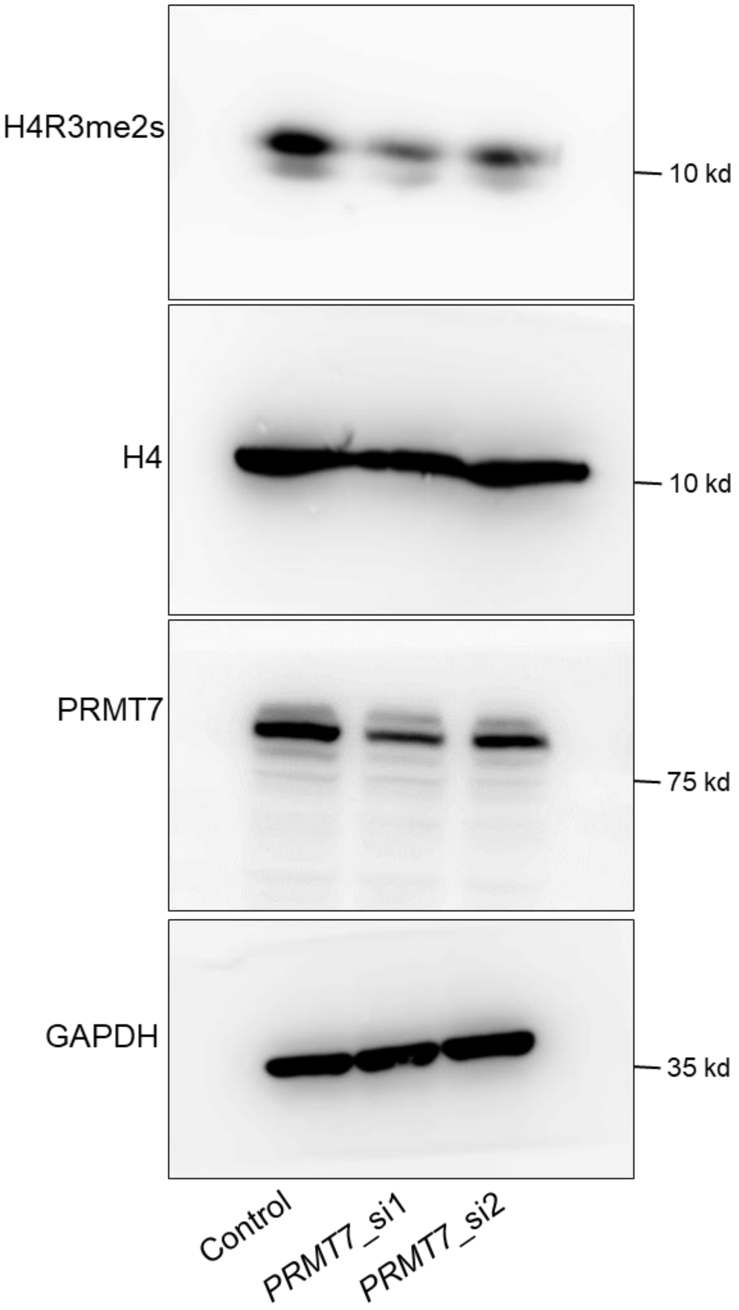

**Supplementary Figure 23**

Supplementary Figure 23a

ct values

| IgG_Input | HNF4A_Input | IgG | HNF4A |
|---|---|---|---|
| 27.49 | 26.88 | 31.87 | 27.04 |
| 27.16 | 26.84 | 32.77 | 26.82 |
| 27.28 | 27.06 | 38.27 | 27.12 |

%Input

| %Input_IgG | %Input_HNF4A |
|---|---|
| 0.0959 | 1.7877 |
| 0.0408 | 2.0346 |
| 0.0684 | 1.9163 |

Supplementary Figure 23b

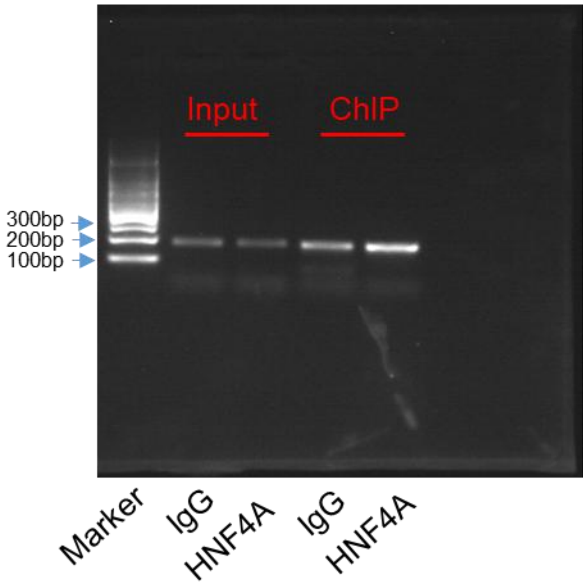


Supplementary Figure 23c

Grey values

| IgG_Input | HNF4A_Input | IgG | HNF4A |
|---|---|---|---|
| 1415 | 1532 | 2865 | 5771 |
| 1363 | 1095 | 3131 | 5879 |
| 1213 | 1197 | 2892 | 5637 |

Relative grey values

| IgG_Input | HNF4A_Input | IgG | HNF4A |
|---|---|---|---|

| | | | |
|---|---|---|---|
| 1.0636 | 1.1516 | 0.9670 | 1.9479 |
| 1.0246 | 0.8231 | 1.0568 | 1.9844 |
| 0.9118 | 0.8998 | 0.9761 | 1.9027 |

**Supplementary Figure 24**

Supplementary Figure 24a

ct values

| IgG_Input | HNF4A_Input | IgG | HNF4A |
|---|---|---|---|
| 26.49 | 26.00 | 30.64 | 26.21 |
| 26.31 | 26.10 | 30.18 | 26.09 |
| 26.01 | 26.14 | 29.60 | 26.15 |

%Input

| %Input_IgG | %Input_HNF4A |
|---|---|
| 0.5633 | 8.6454 |
| 0.6839 | 10.0696 |
| 0.8304 | 9.9309 |

Supplementary Figure 24b

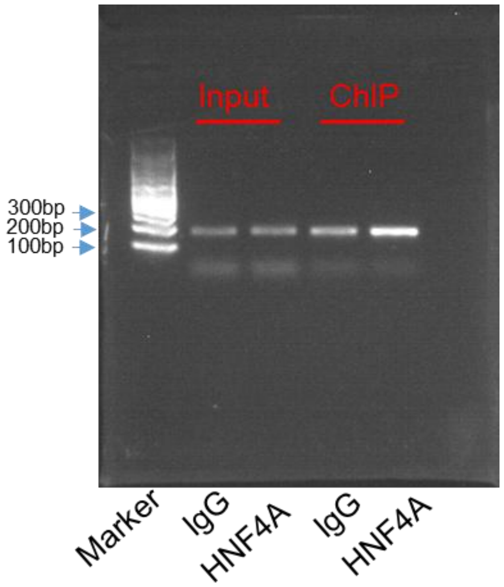


Supplementary Figure 24c

Grey values

| IgG_Input | HNF4A_Input | IgG | HNF4A |
|---|---|---|---|
| 1948 | 2295 | 3520 | 6229 |
| 1742 | 1950 | 3260 | 5950 |
| 1883 | 2124 | 3363 | 6210 |

Relative grey values

| IgG_Input | HNF4A_Input | IgG | HNF4A |
|---|---|---|---|

| | | | |
|---|---|---|---|
| 1.0486 | 1.2354 | 1.0411 | 1.8424 |
| 0.9377 | 1.0497 | 0.9642 | 1.7598 |
| 1.0136 | 1.1434 | 0.9947 | 1.8367 |

**Supplementary Figure 25**

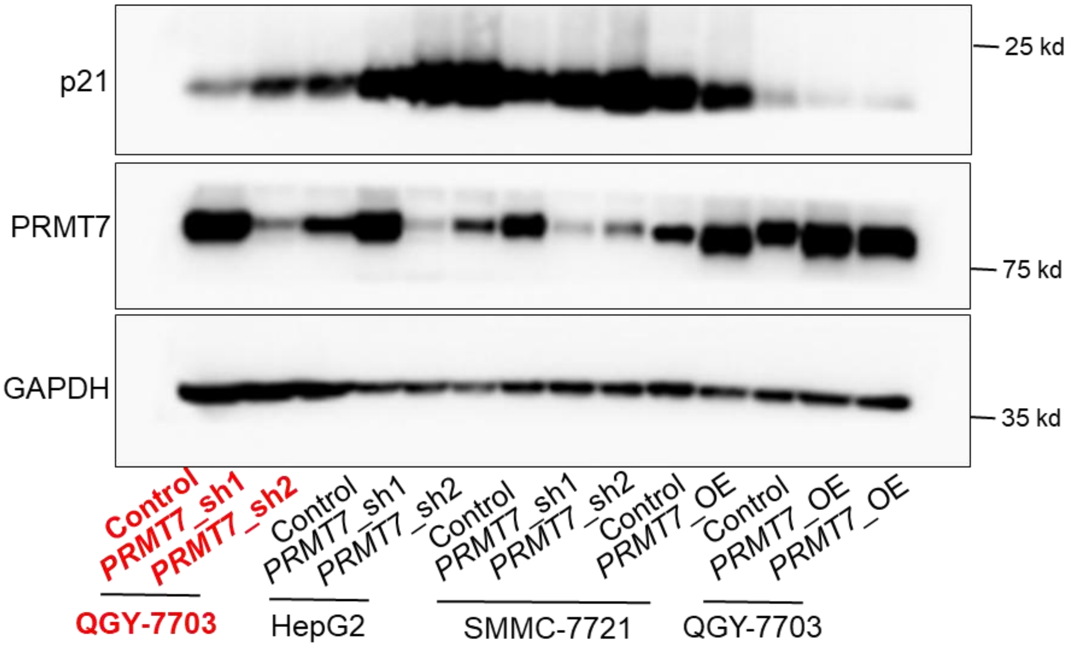

Supplement: Supplementary file 1 — Supplementary Information [file 41467_2022_28861_MOESM1_ESM.pdf]
