# Supplementary material for: An enhancer variant at 16q22.1 predisposes to hepatocellular carcinoma via regulating PRMT7 expression
Source: Nat Commun. 2022 Mar 9;13:1232. doi: 10.1038/s41467-022-28861-0 (PMC8907293; doi:10.1038/s41467-022-28861-0)
Supplement: Supplementary file 2 — Description of Additional Supplementary Files [file 41467_2022_28861_MOESM2_ESM.pdf]

Description of Additional Supplementary Files

File Name: Supplementary Data 1
Description: The results of eQTL analysis for association between rs73613962 and the flanking genes in a one mega-base pair window

File Name: Supplementary Data 2
Description: Primer sequences used in this study

File Name: Supplementary Data 3
Description: Summary statistics for the top 10,000 SNPs in association with HCC risk at the discovery stage
