# Supplementary material for: An enhancer variant at 16q22.1 predisposes to hepatocellular carcinoma via regulating PRMT7 expression
Source: Nat Commun. 2022 Mar 9;13:1232. doi: 10.1038/s41467-022-28861-0 (PMC8907293; doi:10.1038/s41467-022-28861-0)
Supplement: Supplementary file 6 — Reporting Summary [file 41467_2022_28861_MOESM6_ESM.pdf]

nature research

Corresponding author(s): De-Ke Jiang

Last updated by author(s): Nov 25, 2021

Reporting Summary

Nature Research wishes to improve the reproducibility of the work that we publish. This form provides structure for consistency and transparency in reporting. For further information on Nature Research policies, see our Editorial Policies and the Editorial Policy Checklist.

Statistics

For all statistical analyses, confirm that the following items are present in the figure legend, table legend, main text, or Methods section.

n/a	Confirmed	
☐	☒	The exact sample size (n) for each experimental group/condition, given as a discrete number and unit of measurement
☐	☒	A statement on whether measurements were taken from distinct samples or whether the same sample was measured repeatedly
☐	☒	The statistical test(s) used AND whether they are one- or two-sided<br>*Only common tests should be described solely by name; describe more complex techniques in the Methods section.*
☐	☒	A description of all covariates tested
☐	☒	A description of any assumptions or corrections, such as tests of normality and adjustment for multiple comparisons
☐	☒	A full description of the statistical parameters including central tendency (e.g. means) or other basic estimates (e.g. regression coefficient) AND variation (e.g. standard deviation) or associated estimates of uncertainty (e.g. confidence intervals)
☐	☒	For null hypothesis testing, the test statistic (e.g. F, t, r) with confidence intervals, effect sizes, degrees of freedom and P value noted<br>*Give P values as exact values whenever suitable.*
☒	☐	For Bayesian analysis, information on the choice of priors and Markov chain Monte Carlo settings
☒	☐	For hierarchical and complex designs, identification of the appropriate level for tests and full reporting of outcomes
☐	☒	Estimates of effect sizes (e.g. Cohen's d, Pearson's r), indicating how they were calculated

Our web collection on statistics for biologists contains articles on many of the points above.

Software and code

Policy information about availability of computer code

Data collection	No software was used.
Data analysis	For association analysis of SNPs with HCC risk, Plink v1.07 was used. For whole-genome imputation analysis, the Michigan imputation server (https://imputationserver.sph.umich.edu/) was used. For RNA-seq data analysis, FastQC v0.11.8, tophat v2.1.1, GFOLD v1.1.4 softwares were used. The functional enrichment of the differentially expressed genes was analyzed through the Database for Annotation, Visualization and Integrated Discovery (DAVID) v6.8. In addition, ImageJ v1.4.3.67 was used for the analysis of the relative gray value of PCR band.

For manuscripts utilizing custom algorithms or software that are central to the research but not yet described in published literature, software must be made available to editors and reviewers. We strongly encourage code deposition in a community repository (e.g. GitHub). See the Nature Research guidelines for submitting code & software for further information.

Data

Policy information about availability of data

All manuscripts must include a data availability statement. This statement should provide the following information, where applicable:

- Accession codes, unique identifiers, or web links for publicly available datasets
- A list of figures that have associated raw data
- A description of any restrictions on data availability

The RNA-seq data of QGY-7703 cell line before and after PRMT7 knockdown (QGY-7703_control, QGY-7703_PRMT7_sh1, QGY-7703_PRMT7_sh2) have been deposited in the GEO database at NCBI under accession number GSE167432 (https://www.ncbi.nlm.nih.gov/geo/query/acc.cgi). The data used for cis-eQTL in this study are publicly available from the GTEx portal and TCGA portal. GTEx data can be accessed through the GTEx Portal (https://www.gtexportal.org/home), and TCGA data can be accessed through the Genomic Data Commons Data Portal (https://portal.gdc.cancer.gov). KEGG PATHWAY Database can be accessed through KEGG Database (https://www.kegg.jp/kegg/). The ChIP-seq data of three kinds of histone modification (H3K4me3, H3K4me1, and H3K27ac) and transcription factor,

and the data of DNase I hypersensitive site are derived from ENCODE datasets accessed through ENCODE (https://www.encodeproject.org/). HNF4A ChIP-seq data in Caco2 can be accessed in the GEO database at NCBI under accession number GSE23436 (https://www.ncbi.nlm.nih.gov/geo/query/acc.cgi). H4R3me2s ChIP-seq data in mouse embryonic stem cells can be accessed in the GEO database at NCBI under accession number GSE37604 (https://www.ncbi.nlm.nih.gov/geo/query/acc.cgi). Hi-C data in liver can be accessed in the GEO database at NCBI under accession number GSE58752 (https://www.ncbi.nlm.nih.gov/geo/query/acc.cgi). Source data are provided with this paper. All other data are available from the corresponding author upon request.

Field-specific reporting

Please select the one below that is the best fit for your research. If you are not sure, read the appropriate sections before making your selection.

[x] Life sciences [ ] Behavioural & social sciences [ ] Ecological, evolutionary & environmental sciences

For a reference copy of the document with all sections, see nature.com/documents/nr-reporting-summary-flat.pdf

Life sciences study design

All studies must disclose on these points even when the disclosure is negative.

Sample size	To genome-wide survey of HCC risk-associated enhancer variant, this study included 4,898 HCC cases and 7,060 non-HCC controls, which has >95% statistical power to detect associations for SNPs with risk allele frequency of >0.05 and with OR of >1.4.
Data exclusions	At the discovery stage for genome-wide survey of HCC risk-associated enhancer variant, SNPs with a minor allele frequency less than 0.05 or significantly deviated from the Hardy-Weinberg equilibrium (P < 0.0001) were removed from further analyses. Since only common SNPs with high quality are considered in this study.
Replication	To validate the SNPs screened out from the discovery stage, we took out two round replications, with four independent populations. In each population, rs73613962 was consistently validated to be significantly associated with HCC risk (P < 0.05 in each subject set, and with the same direction of association as in the discovery stage).
Randomization	Not relevant to this study as this is a genetic epidemiology and functional experiment study. We carefully controlled for relevant covariates in relevant analyses.
Blinding	Not relevant to this study as this is a genetic epidemiology and functional experiment study.

Reporting for specific materials, systems and methods

We require information from authors about some types of materials, experimental systems and methods used in many studies. Here, indicate whether each material, system or method listed is relevant to your study. If you are not sure if a list item applies to your research, read the appropriate section before selecting a response.

Materials & experimental systems

n/a	Involved in the study	
[ ]	[x]	Antibodies
[ ]	[x]	Eukaryotic cell lines
[x]	[ ]	Palaeontology and archaeology
[ ]	[x]	Animals and other organisms
[ ]	[x]	Human research participants
[x]	[ ]	Clinical data
[x]	[ ]	Dual use research of concern

Methods

n/a	Involved in the study	
[x]	[ ]	ChIP-seq
[x]	[ ]	Flow cytometry
[x]	[ ]	MRI-based neuroimaging

Antibodies

Antibodies used	PRMT7 (Abcam, cat. no. ab179822; Abcam, cat. no. ab181214), H4R3me2s (Active motif, cat.no. 61187), H4 (Abcam, cat. no. ab177840), p21 (CST, cat. no. 2947S), GAPDH (CST, cat. no. 2118S), anti-Rabbit IgG (HRP conjugate, YEASON), HNF4A (Abcam, cat.no. ab181604).
Validation	The all antibodies used in this study were validated in human cell lines QGY-7703, HepG2 and 293T. PRMT7 (Abcam, cat. no. ab179822), H4R3me2s (Active motif, cat.no. 61187), H4 (Abcam, cat. no. ab177840), p21 (CST, cat. no. 2947S), GAPDH (CST, cat. no. 2118S), anti-Rabbit IgG (HRP conjugate, YEASON) were applied to western blot. PRMT7 (Abcam, cat. no. ab181214) was applied to immunohistochemistry (IHC) assay. HNF4A antibody was applied to chromatin immunoprecipitation (ChIP) and electrophoretic mobility shift assay (EMSA). H4R3me2s (Active motif, cat.no. 61187) was applied to ChIP.

Eukaryotic cell lines

Policy information about cell lines

Cell line source(s)	QGY-7703, HepG2, SMMC-7721, HepAD38, SNU398, and 293T cell lines were originally purchased from the Cell Bank of the Chinese Academy of Sciences (Shanghai, China).
Authentication	All cell lines were authenticated by short-tandem-repeat (STR) profiling.
Mycoplasma contamination	All cell lines tested were negative for mycoplasma contamination.
Commonly misidentified lines (See ICLAC register)	No commonly misidentified cell lines were used in this study.

Animals and other organisms

Policy information about studies involving animals; ARRIVE guidelines recommended for reporting animal research

Laboratory animals	The nude mice we used were four-weeks old female BALB/c nude mice. These mice were housed in the specific pathogen-free (SPF) environment at a constant temperature (26°C~28°C) and a relatively constant humidity (40 ~ 60%), with 10 h light/14 h dark cycle.
Wild animals	No wild animals were used.
Field-collected samples	No field-collected samples were used.
Ethics oversight	All animal experiments were approved by the Ethical Committee for Experimental Animal Care of Nanfang Hospital, Southern Medical University (Guangzhou, China).

Note that full information on the approval of the study protocol must also be provided in the manuscript.

Human research participants

Policy information about studies involving human research participants

Population characteristics	In the discovery stage, 2,514 chronic HBV carriers were included, consisting of 1,161 HCC cases and 1,353 non-HCC controls from East China (Qidong, Jiangsu province). In the replication stage 1, a total of 1,297 chronic HBV carriers including 576 HCC cases and 721 non-HCC controls were enrolled from East China (Shanghai). In the replication stage 2, a total of 8,147 chronic HBV carriers including three independent subject sets (the replication stage 2a: 1,942 HCC cases and 2,812 non-HCC controls; the replication stage 2b: 393 HCC cases and 1,314 non-HCC controls; the replication stage 2c: 826 HCC cases and 860 non-HCC controls) were recruited from East China (Shanghai and Jiangsu province), North China (Beijing and Shandong province), and South China (Guangxi province), respectively. In these five independent populations, both the majority of cases and controls are male, which range from 77.3% to 84.7% in cases and 59.9% to 78.8% in controls. The mean ages of the cases at enrollment in these five independent populations were about 50 years, ranging from 47.8 to 54.4, while the mean ages of the controls at enrollment in these five independent populations were also about 50 years, ranging from 46.1 to 53.0, except for the controls in the replication stage 2b (33.6).
Recruitment	All the HCC cases and non-HCC controls in the discovery stage were recruited by Qidong Liver Cancer Institute in Qidong County, Jiangsu Province, China, during the period from May 2006 to December 2012. All the subjects in the replication stage 1 were recruited from the affiliated hospitals of the Second Military Medical University, Shanghai, China, from October 2009 to September 2011. The study subjects recruited in the replication stage 2a were diagnosed, hospitalized and treated in the affiliated hospitals of Soochow University or the Suzhou Municipal Hospital from 2007 to 2010. The subjects in the replication stage 2b were recruited from Beijing Ditan Hospital and Beijing You'an Hospital during the period of November 2001 to August 2004. The subjects in the replication stage 2c were enrolled by Youjiang Medical College for Nationalities during January 2004 and December 2010. All the subjects involved in this study are chronic HBV carriers defined as individuals that is positive for both HBV surface antigen (HBsAg) and immunoglobulin G antibody to HBV core antigen for at least 6 months. All the cases are HCC patients and all the controls are non-HCC chronic hepatitis B patients. Diagnosis with HCC was based on (i) positive findings on cytological or pathological examination and/or (ii) positive images on angiogram, ultra-sonography,computed tomography and/or magnetic resonance imaging, combined with an α-fetoprotein concentration of ≥400 ng/ml. We confirmed that none of the individuals with HCC had other cancers through an initial screening. All controls had, by self-report, no history of HCC or other cancers. All the cases and controls in each population were recruited from the same area of China, which were expected to have similar genetic backgrounds, thus reducing the interference of genetic confounding factors in this study.
Ethics oversight	Informed consent was obtained from all study subjects before their participation in the study. The study was approved by the ethical committees of all institutions involved in the study, they are the Ethics Committee of Qidong Liver Cancer Institute, the Ethics Committee of the Second Military Medical University, the Ethical Committee of Soochow University, the Ethical Committees of Beijing Ditan Hospital and Beijing You'an Hospital, and the Ethic Committee of Youjiang Medical College for Nationalities. This study was conducted in accordance with Declaration of Helsinki principles and compliant with the "Guidance of the Ministry of Science and Technology (MOST) for the Review and Approval of Human Genetic Resources".

Note that full information on the approval of the study protocol must also be provided in the manuscript.
